# Caspase-8 in inflammatory diseases: a potential therapeutic target

**DOI:** 10.1186/s11658-024-00646-x

**Published:** 2024-10-08

**Authors:** Wangzheqi Zhang, Chenglong Zhu, Yan Liao, Miao Zhou, Wenyun Xu, Zui Zou

**Affiliations:** 1 Faculty of Anesthesiology, Changhai Hospital, Naval Medical University, Shanghai, 200433 China; 2 Department of Anesthesiology, The Affiliated Cancer Hospital of Nanjing Medical University, Jiangsu Cancer Hospital, Jiangsu Institute of Cancer Research, Nanjing Medical University, Nanjing, 210009 Jiangsu China; 3 Department of Anesthesiology, Second Affiliated Hospital of Naval Medical University, Shanghai, 200003 China

**Keywords:** Caspase-8, Apoptosis, Necroptosis, Pyroptosis, PANoptosis, Inflammatory disease

## Abstract

Caspase-8, a renowned cysteine-aspartic protease within its enzyme family, initially garnered attention for its regulatory role in extrinsic apoptosis. With advancing research, a growing body of evidence has substantiated its involvement in other cell death processes, such as pyroptosis and necroptosis, as well as its modulatory effects on inflammasomes and proinflammatory cytokines. PANoptosis, an emerging concept of cell death, encompasses pyroptosis, apoptosis, and necroptosis, providing insight into the often overlapping cellular mortality observed during disease progression. The activation or deficiency of caspase-8 enzymatic activity is closely linked to PANoptosis, positioning caspase-8 as a key regulator of cell survival or death across various physiological and pathological processes. Aberrant expression of caspase-8 is closely associated with the development and progression of a range of inflammatory diseases, including immune system disorders, neurodegenerative diseases (NDDs), sepsis, and cancer. This paper delves into the regulatory role and impact of caspase-8 in these conditions, aiming to elucidate potential therapeutic strategies for the future intervention.

## Introduction

Cysteine-aspartic proteases, commonly known as caspases, are a family of proteases characterized by their specificity for cysteine at their active sites and their ability to cleave after aspartic acid residues [1]. These proteases are typically present in cells as inactive zymogens (procaspases) and become biologically active upon conversion to their active form. Due to their specificity, caspases selectively cleave certain proteins at specific sites, triggering the activation or inactivation of downstream proteins. This evolutionarily conserved family of proteases whose dysregulation in activity or expression levels is intimately linked to the pathogenesis of numerous human diseases, such as cancer, autoimmune disorders, and neurodegenerative diseases (NDDs). Research indicates that modulation of caspase activity can significantly ameliorate disease progression [2].

Caspases are primarily responsible for regulating programmed cell death (PCD), such as apoptosis, and play a crucial role in modulating immune and inflammatory responses by activating cytokines and cell death-related signaling pathways. According to their functions, caspases are categorized as either apoptotic (caspase-2, -3, -6, -7, -8, -9, and -10) or inflammatory (caspase-1, -4, -5, and -11). Apoptotic caspases are further divided into initiator and executioner caspases. Initiator caspases, including caspase-2, -8, -9, and -10, can initiate apoptosis by self-activation and regulating effector caspases. Executioner caspases, such as caspase-3, -6, and -7, are responsible for cleaving cellular proteins and executing the apoptotic process. Upon stimulation by extrinsic signaling proteins, initiator caspases are activated, leading to the cleavage and activation of downstream effector caspases. The activated executioner caspases then hydrolyze target proteins, thereby initiating the apoptotic process [3–5].

Inflammatory caspases are activated by inflammasomes, resulting in pyroptotic cell death and the production of inflammatory mediators. These mediators subsequently trigger both protective and pathological immune responses. The N-terminal regions of initiator and inflammatory caspases contain a caspase recruitment domain (CARD; caspase-1, -2, -4, -5, -9, -11) or two death effector domains (DEDs; caspase-8, -10), which facilitates their recruitment and activation within protein complexes [1].

Caspase-8 is a prototypical initiator caspase, encoded by the CASP8 gene and known as MACH (MORT1-associated CED-3 homolog), FLICE (FADD-like ICE), or Mch5 (mammalian Ced homolog 5). First cloned in 1996 by Muzio et al., the precursor of caspase-8, known as procaspase-8 or caspase-8 zymogen, consists of 479 amino acids. Caspase-8 is a crucial enzyme in the apoptotic pathway, and its structure and function are highly conserved across mammalian species, including humans. Its N-terminal domain contains two DEDs (DED1 and DED2), each approximately 70 amino acids in length, while its C-terminal domain includes a large protease subunit (p20/p18) with an active catalytic cysteine and a small protease subunit (p12/10) with a substrate-binding domain; the large and small subunits are constituted by Ser217-Asp374 and Asp384-Asp479 segments, respectively, giving caspase-8 a molecular weight of 55 kD [6–9].

Caspase-8 plays pivotal roles in various physiological and pathological functions in the human body, most notably in regulating apoptosis via the extrinsic death receptor pathway. Recent studies have also revealed its importance in pyroptosis and necroptosis, as well as in immune and inflammatory processes. Dysregulation of caspase-8 and its mediated signaling pathways is associated with various diseases in humans, including inflammatory bowel disease (IBD), autoimmune diseases, and cancer [10–12]. As a pivotal molecule, caspase-8 mediates and participates in signaling pathways involved in various pathophysiological processes, functioning as a “molecular switch” that influences multiple diseases and aspects of human health.

Understanding apoptosis and related processes has always been fundamental to cellular biology. The association between these processes and inflammation, particularly through molecules like caspase-8, has emerged as a critical area of research. This is due to the increasing recognition of inflammation’s role in a wide range of diseases, from autoimmune diseases to cancer. Our review underscores the relevance of caspase-8 within this context, highlighting its potential as a therapeutic target.

### Caspase-8 and apoptosis

Current research unequivocally establishes that caspase-8 stands as the most pivotal initiator caspase in the cell death pathways mediated by death receptors (DRs) such as FAS, TNFR1, or DR4. Caspase-8, a crucial mediator of extrinsic apoptosis, is activated by the death-inducing signaling complex (DISC), demonstrating its functional efficacy. Within this assembly, FAS discerns external signals by binding with the FAS ligand (FASL) to convey the pro-apoptotic signals. FAS-associated protein with death domain (FADD), an adapter protein, is characterized by a C-terminal death domain (DD) and an N-terminal DED. Upon signal reception, FAS recruits and binds FADD through homotypic DD interactions. This chain of events then prompts a conformational change in the DED of FADD after DR binding, enabling it to engage with the DED1 of cytosolic procaspase-8, culminating in the formation of DISC [7, 13, 14]. Within the DISC, procaspase-8 molecules aggregate and undergo self-activation, generating the active caspase-8, which initiates apoptosis by cleaving and activating executioner proteins such as caspase-3 and caspase-7 to execute apoptotic effects [5]. Beyond the FAS receptor, TNFR1 can also mediate apoptosis via caspase-8, albeit through a more intricate mechanism. During TNFR1’s orchestration of the apoptotic pathway, tumor necrosis factor receptor type 1-associated death domain protein (TRADD) is activated first and subsequently recruits FADD. Besides inducing apoptosis, it can also trigger the expression of cFLIP through the NF-κB pathway, thus inhibiting apoptosis. Within this process, receptor-interacting protein kinase 1 (RIPK1) emerges as a critical molecule. Post-translation, its ubiquitination and phosphorylation status determine cellular fate—survival or death. Once recruited by adapter protein TRADD, RIPK1 undergoes extensive ubiquitination by cellular inhibitors of apoptosis (cIAP) 1 and 2, activating NF-κB and MAPK pathways, releasing pro-inflammatory cytokines and promoting an anti-apoptotic response. In this process, TRADD and RIPK1 may engage in competitive interactions. Under specific conditions, if RIPK1 is not effectively recruited or its expression is suppressed, the apoptotic pathway is inhibited, thereby enabling the cell to evade programmed cell death [15–17]. In contrast, when RIPK1 is deubiquitinated by “death signal” cylindromatosis (CYLD) [18–22], it associates with FADD and procaspase-8 to form RIPK1-FADD-caspase-8 complex (complex IIa), leading to cellular death [14, 23].

In the context of apoptosis, cellular FLICE-inhibitory protein (cFLIP) plays a decisive role. This catalytically inactive homolog of caspase-8 modulates caspase-8 activity by binding to it [10]. cFLIP exists in two primary isoforms: the long variant (cFLIP_L_) and short variant (cFLIP_S_), with different structure and function. Comprising a long C-terminus, two DEDs, and a caspase-like domain, cFLIP_L_ closely resembles caspase-8, forming heterodimers with procaspase-8, but lacks the complete active site necessary for substrate cleavage. The roles of cFLIP_L_ are complex and not fully understood, but it is generally believed to have a dual influence on caspase-8, enhancing apoptosis at lower concentrations while inhibiting it at higher concentrations [14]. At low levels, procaspase-8 is prone to heterodimerization with cFLIP_L_ rather than homodimerization. Such heterodimers, with their DED chains akin to those on procaspase-8 homodimers, can activate procaspase-8, potentially promoting apoptotic signaling at lower levels [6, 7, 13, 24]. Conversely, cFLIP_S_, composed of two DEDs and a truncated C-terminus lacking a complete Caspase-like domain, can competitively inhibit DED-mediated DISC recruitment of procaspase-8, thereby blocking the activation of caspase-8 and preventing the initiation of apoptosis [6, 8, 25–28]. For the aforementioned phenomenon, the traditional competitive model posits that cFLIP competes with caspase-8 for binding to FADD, thereby inhibiting the recruitment and activation of caspase-8. However, the co-operative and hierarchical binding model proposed by Michelle A et al. [7] suggests that procaspase-8 binds to the FL motif of FADD through its DED1 hydrophobic pocket, and subsequently interacts with the DED1 of cFLIP_L/S_ via the FL motif of its DED2, forming procaspase-8: cFLIP_L/S_ heterodimers. The composition of these heterodimers ultimately determines the activity of caspase-8. Notably, cFLIP_S_ is unable to form death effector filaments, thus preventing the oligomerization of procaspase-8 and inhibiting the activation of caspase-8. In cells overexpressing cFLIP_S_, the ratio of FADD: caspase-8: cFLIP approaches 1:1:1, indicating that cFLIP_S_ can alter the composition of the DISC and inhibit caspase-8 activity. In summary, the interaction between cFLIP and caspase-8 represents a complex regulatory process that collectively determines cell fate. The co-operative and hierarchical binding model elucidates the dual functions of cFLIP_L_ and reveals the unique mechanism by which cFLIP_S_ inhibits caspase-8 [7, 13]. Thus, the isoforms and levels of cFLIP are critical in regulating apoptosis, a process essential for growth and development as it can eliminate potential pathogenic cells, such as inflamed or damaged cells, thereby maintaining homeostasis in the body.

**Fig. 1:**
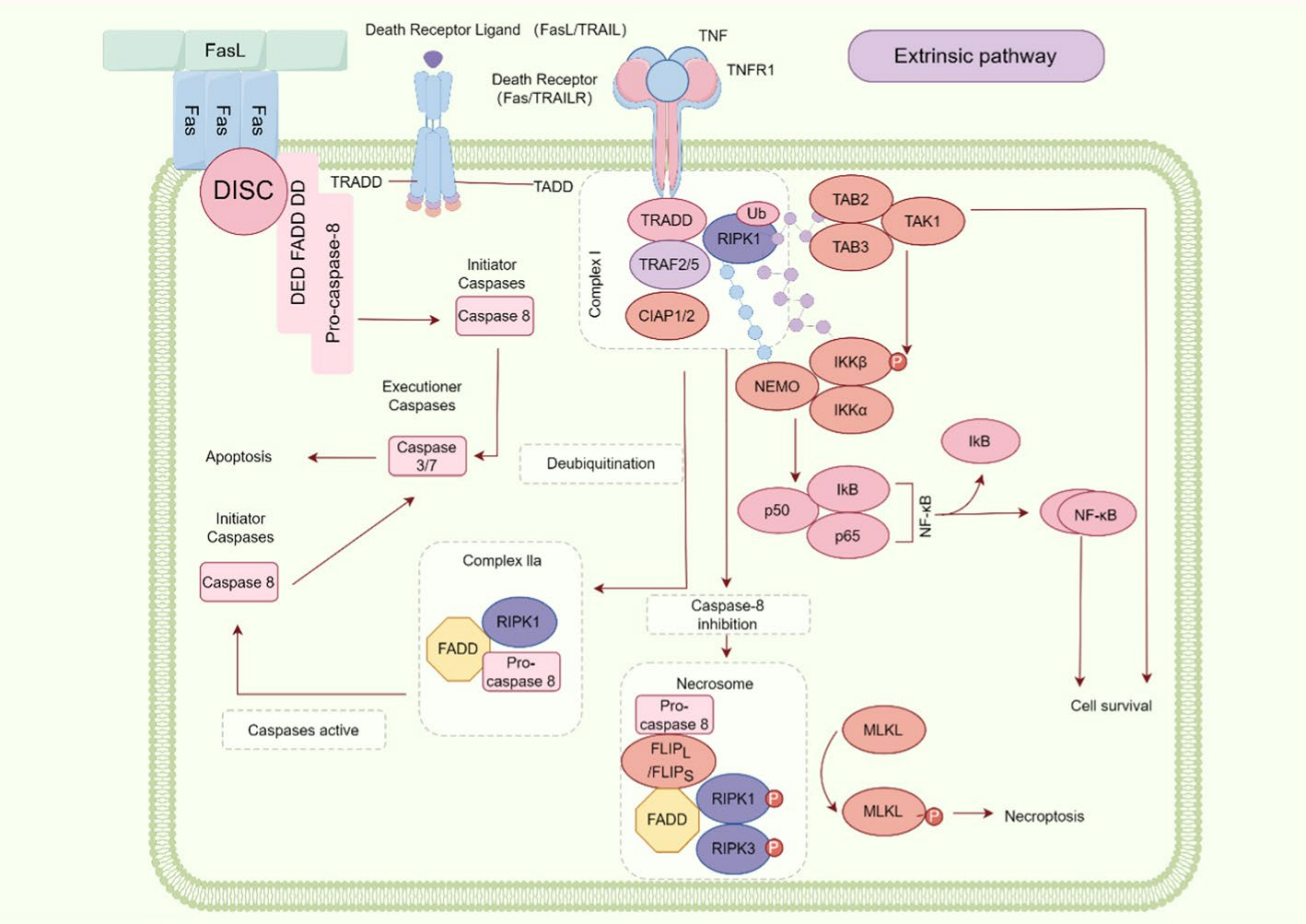
Schematic representation of caspase-8 mediated extrinsic apoptosis. Caspase-8 is activated by the death-inducing signaling complex (DISC), subsequently cleaving effector proteins caspase-3 and caspase-7, thereby initiating apoptosis. In the apoptosis pathway activated by tumor necrosis factor receptor 1 (TNFR1), the ubiquitination status of receptor-interacting protein kinase 1 (RIPK1) is a critical determinant of cell fate, dictating survival or death. Moreover, when the activity of caspase-8 is inhibited, the apoptotic pathway is suppressed, potentially triggering necroptotic cell death (by Figdraw)

In summary, caspase-8 serves as a pivotal initiator of the extrinsic apoptosis pathway, activated through the DISC upon death receptor engagement. This protease not only triggers a cascade involving effector caspases like caspase-3 and caspase-7 but also intricately interacts with regulatory proteins such as cFLIP and RIPK1, modulating the balance between cell survival and apoptosis. Uniquely, the dual roles of cFLIP isoforms highlight a sophisticated regulatory mechanism where lower cFLIP_L_ levels enhance caspase-8 activation, while higher levels inhibit it. This nuanced regulation, coupled with the interplay of ubiquitination and deubiquitination processes involving RIPK1, underscores the essential role of caspase-8 in apoptosis. From a novel perspective, targeting the modulatory interactions of caspase-8 with cFLIP and RIPK1 presents promising therapeutic potential for conditions characterized by aberrant apoptotic signaling. The role of caspase-8 in extrinsic apoptosis is illustrated in Fig. 1.

### Caspase-8 and necroptosis

Beyond the well-established role of caspase-8 in apoptosis, recent discoveries have highlighted its involvement in the regulation of necroptosis. Historically, necrosis was postulated as an uncontrolled form of cell death, resulting from accidental demise due to extrinsic environmental insults [29–31]. However, recent insights have revealed a form of cell death, necroptosis, which shares similarities with both apoptosis and necrosis but remains distinct. Morphologically akin to necrosis, it is characterized by cellular swelling, organelle distension, and plasma membrane rupture [31], yet it is differentiated by its programmatic regulation by genetic components, setting it apart from necrosis. Dependent on the interplay and modulation of various proteins, it is now understood that the extrinsic apoptotic and necroptotic pathways are intricately intertwined, with caspase-8 at the center of this interaction [14]. Necroptosis can be activated by receptors such as tumor necrosis factor receptor 1 (TNFR1), DRs like TNFR and FAS, and Toll-like receptors 3/4 (TLR3/4) [32–38], with the signaling cascade proceeding through the RIPK1-RIPK3-MLKL pathway. As previously mentioned, upon receptor activation, active RIPK1 is recruited to a complex containing FADD and caspase-8 (complex IIa). In the absence of active caspase-8, RIPK3 is recruited and phosphorylated by RIPK1, forming a structure known as the ripoptosome [37–40], which then recruits and phosphorylates MLKL, assembling the necrosome and mediating necrotic cell death [38, 41–44]. Moreover, once the ripoptosome is activated, RIPK3 can phosphorylate the mitochondrial pyruvate dehydrogenase complex (PDC), enhancing aerobic respiration and mitochondrial ROS production [37]. Numerous studies [35, 45, 46] corroborate that necroptosis can occur independently of RIPK1, though RIPK3 and MLKL are still key players. This finding challenges the conventional view of RIPK1 as an indispensable initiator of necroptosis, suggesting alternative pathways or regulatory mechanisms. The independence of necroptosis from RIPK1 implies a more robust and adaptable cell death mechanism, potentially involving other kinases or cellular stress responses that can directly activate RIPK3.

Caspase-8 has been shown to cleave key proteins involved in necroptosis, including RIPK1 [47], RIPK3 [48], and CYLD [49]. This cleavage activity serves as a critical regulatory mechanism, inhibiting necroptosis and promoting apoptosis, thereby modulating the inflammatory and tumorigenic microenvironment [6]. The pivotal role of caspase-8 in this process underscores its importance as a determinant of cellular fate and the subsequent inflammatory outcomes. Apoptosis and necroptosis function as opposing forces within the cellular equilibrium, dictating the fate and inflammatory response of cells under stress or injury. Apoptosis, characterized by the orderly dismantling of cellular components into apoptotic bodies, typically results in an anti-inflammatory environment, as these bodies are efficiently phagocytosed without triggering an inflammatory response in neighboring cells. In contrast, necroptosis leads to cell death through plasma membrane disruption, releasing intracellular contents that provoke a robust inflammatory reaction in adjacent cells, thereby escalating the overall inflammatory response [14]. The dynamic interplay between apoptosis and necroptosis, mediated by caspase-8, suggests a sophisticated cellular strategy to adapt to various pathological conditions. By controlling the balance between these two cell death pathways, cells can tailor their response to different stressors, optimizing survival or triggering death in a context-dependent manner.

The intricate interplay between caspase-8 and RIPK1 has emerged as a pivotal node in the regulation of inflammatory responses and cell death signaling. Evidence suggests that caspase-8-mediated cleavage of RIPK1 serves not only as a checkpoint in apoptosis but also a critical mechanism for maintaining inflammatory homeostasis. A study by Panfeng et al. highlighted the importance of this mechanism, demonstrating that its inhibition can lead to an autoinflammatory state with heightened sensitivity to both apoptotic and necrotic stimuli [50–53]. However, the regulation of necroptosis is a multifaceted process, and while the cleavage of RIPK1 and RIPK3 is significant, it is not the sole determinant [9, 37, 54, 55]. A novel perspective is introduced by the observation that mutations at the RIPK1 cleavage site can lead to embryonic lethality in mice, a phenotype that cannot be rescued by the absence of RIPK3 or MLKL. This finding indicates that the interaction between caspase-8 and RIPK1 is essential for the survival of developing organisms and that the balance between apoptosis and necroptosis is delicately maintained. Caspase-8-mediated cleavage of RIPK1 acts as a central hub, coordinating the suppression of both apoptotic and necroptotic pathways. Concurrent inhibition of FADD-caspase-8-mediated apoptosis and RIPK3–MLKL-mediated necroptosis is necessary to prevent mortality in mice, signifying a dual role for caspase-8 in regulating these pathways [31, 50, 51, 53]. These insights suggest a potential therapeutic strategy where modulating the caspase-8/RIPK1 axis could provide a dual mechanism of action against diseases characterized by dysregulated cell death and inflammation. In summary, the caspase-8/RIPK1 axis represents a critical junction in the regulation of cell death and inflammation. A nuanced understanding of this axis not only illuminates the fundamental mechanisms of cell death but also opens new avenues for developing targeted therapeutics. Future research should strive to elucidate the precise molecular underpinnings of this axis and explore its therapeutic potential in vivo, which could pave the way for novel precision medicine treatments for inflammatory and degenerative diseases.

**Fig. 2:**
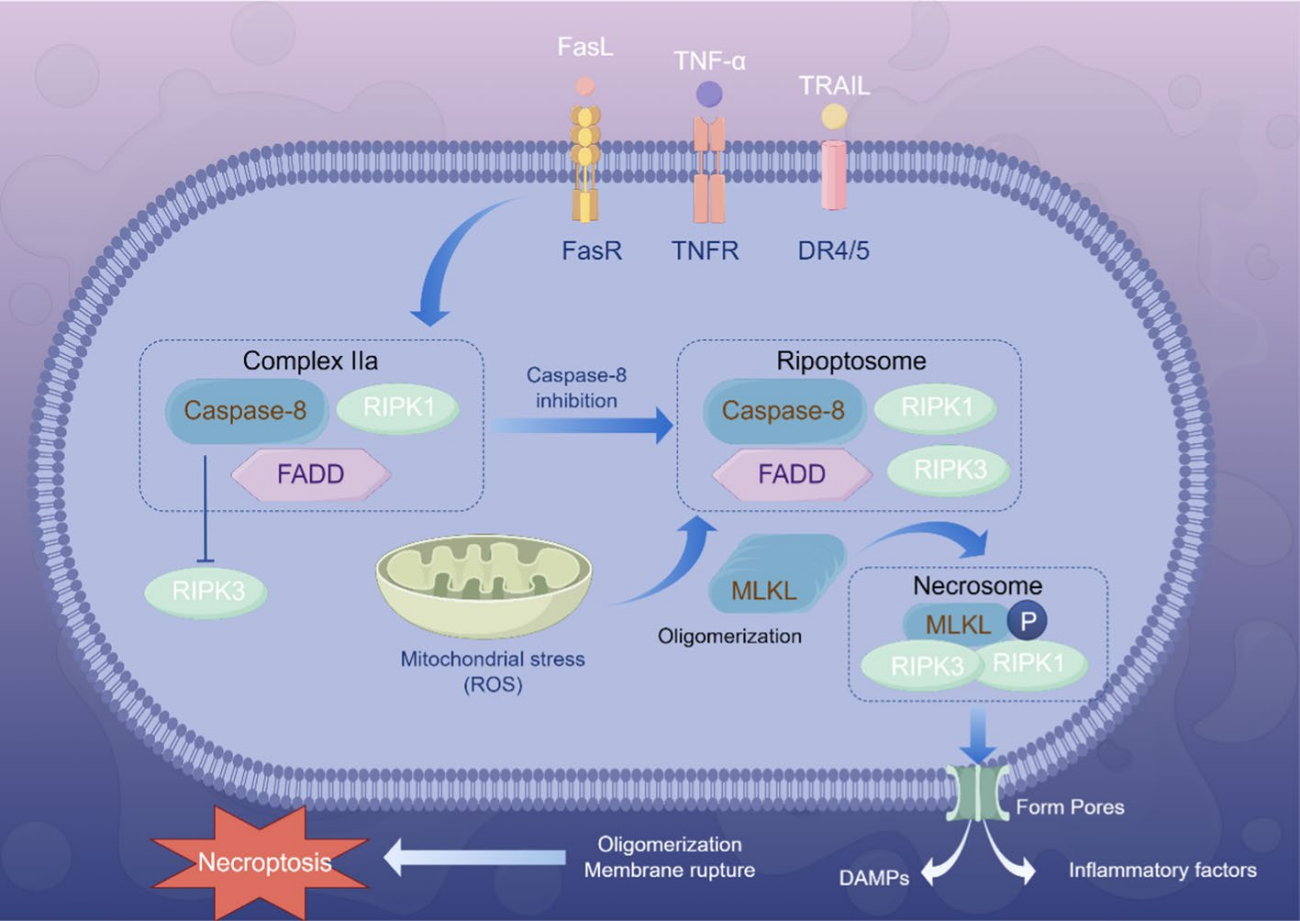
The role of caspase-8 in necroptosis. Necroptosis can be initiated by receptors such as the Fas receptor (FasR), TNF receptor (TNFR), and other death receptors (DRs), and proceeds via the receptor-interacting protein kinase 1 (RIPK1)–receptor-interacting protein kinase 3 (RIPK3)–mixed lineage kinase domain-like protein (MLKL) signaling axis. Following receptor activation, active RIPK1 is co-opted into a complex comprising Fas-associated death domain (FADD) and caspase-8 (complex IIa). When caspase-8 activity is inhibited, RIPK3 is then recruited and phosphorylated by RIPK1, leading to the formation of a complex known as the ripoptosome. The ripoptosome subsequently recruits and phosphorylates MLKL to assemble the necrosome, which ultimately orchestrates necroptotic cell death (by Figdraw)

In conclusion, caspase-8 is integral to the regulation of necroptosis through the cleavage of essential proteins, including RIPK1, RIPK3, and CYLD. This proteolytic activity inhibits the necroptotic pathway but facilitates the induction of apoptosis. This regulatory function is crucial for maintaining the balance between apoptosis and necroptosis, thereby influencing the inflammatory and tumorigenic microenvironment. The innovative perspective on caspase-8’s role suggests that it serves as not only a gatekeeper between these two cell death pathways but also potentially modulates other cellular functions such as inflammatory homeostasis and apoptotic signaling. This multifaceted role of caspase-8 underscores its significance in cellular fate determination and highlights the potential for targeted therapeutic interventions aimed at manipulating caspase-8 activity to manage pathological conditions characterized by dysregulated necroptosis. The role of caspase-8 in necroptosis is depicted in Fig. 2.

### Caspase-8 and pyroptosis

Although pyroptosis is recognized as a form of PCD distinct from necroptosis, it was traditionally considered a response to certain bacterial damage factors, pathogens, and damage-associated molecular patterns (DAMPs), primarily mediated by caspase-1 [6, 56]. However, recent discoveries have unveiled that other members of the caspase family can also contribute to the pyroptosis process, and there even exist caspase-independent pathways (such as those involving granzyme A or B) [57, 58]. Pyroptosis often occurs following infection by pathogens, suggesting its probable role as part of an anti-infection or antimicrobial response. This process can release a plethora of cytokines to recruit immune cells, amplifying the inflammatory response to combat infections or cancer [59, 60]. Upon pyroptosis, cell membranes rupture, cells swell, chromatin condenses, and intracellular contents are released, thereby summoning more inflammatory factors, exacerbating the inflammatory response and leading to tissue damage. However, unlike necrosis, the integrity of the nucleus and mitochondria remains intact during pyroptosis [37]. Traditional pyroptosis pathways are categorized into the caspase-1-mediated canonical pathway and the caspase-4/5/11-mediated noncanonical pathway. The canonical pathway involves the activation of procaspase-1 by pattern recognition receptors (PRRs), including the nucleotide-binding oligomerization domain-like receptors (NLRs) family, the DNA receptor absent in melanoma 2 (AIM2), and the pyrin receptor [36], upon detecting invasive microbial signals or pathogen-associated molecular patterns (PAMPs) and intrinsically released DAMPs. This subsequently leads to the release of inflammatory cytokines and cleavage of gasdermin D (GSDMD), inducing pore formation in the cell membrane, which causes a massive release of inflammatory substances and triggers a cascading inflammatory storm [61–63]. The noncanonical pathway, mediated by human caspase-4/5 and mouse caspase-11, can directly recognize and oligomerize bacterial lipopolysaccharides (LPS) and cleave GSDMD, promoting pore formation in cell membranes and inducing pyroptosis through the release of IL-1α [64–67]. Beyond these pathways, researchers discovered a caspase-3-dependent pyroptosis pathway in 2017, the caspase-3-GSDME pathway [68]. Further exploration into this novel target revealed that caspase-3 can recognize and cleave the N-terminal domain of GSDMD, thereby exerting an inhibitory effect on pyroptosis [69], highlighting the complexity of the pyroptosis process.

Previously, caspase-8 was primarily considered involved in apoptosis rather than pyroptosis [6]. However, recent studies indicate that caspase-8 can mediate an alternative pathway independent of caspase-1 and caspase-4/5/11. It shares the same cleavage site on GSDMD with caspase-1, albeit with significantly lower efficiency. In the absence of caspase-1 or the pyroptotic effector GSDMD, inflammasomes can activate caspase-8, leading to a delayed, alternative secondary pyroptosis. This might serve as a protective mechanism against infections, with GSDMD-dependent pyroptosis inhibiting the activation of caspase-8 within the inflammasome, thus blocking the aforementioned process [70]. Danielle et al. found that in the presence of *Legionella pneumophila* flagellin, the binding and assembly of neuronal apoptosis inhibitory protein 5 (NAIP5), NLR family CARD-containing protein 4 (NLRC4), and apoptosis-associated speck-like protein containing a CARD (ASC), could recruit and activate caspase-8 in the absence of caspase-1 or GSDMD, inducing cell death. This mechanism might serve as a reserve strategy for cell death, aiming to limit bacterial replication or macrophage functionality [71]. In the study conducted by Fritsch et al. [68], it was elucidated that in murine models, mutating the caspase-8 Cys362 (C362) residue to C362S abrogates its catalytic activity and plays a pivotal role in triggering caspase-1-mediated pyroptosis. The expression of CASP8^C362S^ catalyzes the formation of ASC specks, activation of caspase-1, and secretion of IL-1β, suggesting that catalytically inactive caspase-8, serving as a molecular scaffold, is crucial for the activation of inflammasomes and the pyroptosis process when both apoptosis and necroptosis are inhibited [72]. Furthermore, in the presence of caspase-1, the DED of caspase-8 can interact with the pyrin domain (PYD) of ASC, contributing to the activation of the NLRP3-dependent, caspase-1-mediated pyroptosis pathway. This interaction facilitates the formation of the IL-1 and NLRP3 inflammasomes [73, 74]. Additionally, research has revealed that caspase-8 can act as an upstream protein to activate caspase-3, which in turn cleaves GSDME to regulate pyroptosis, or directly cleave GSDMD or GSDME to induce pyroptosis [75–77]. In summary, although the research on caspase-8 in pyroptosis is still in its early stages, it is clear that caspase-8 is a critical determinant in the interplay and influence among apoptosis, necroptosis, and pyroptosis, serving as a key regulator of cellular homeostasis and determines cell fate.

**Fig. 3:**
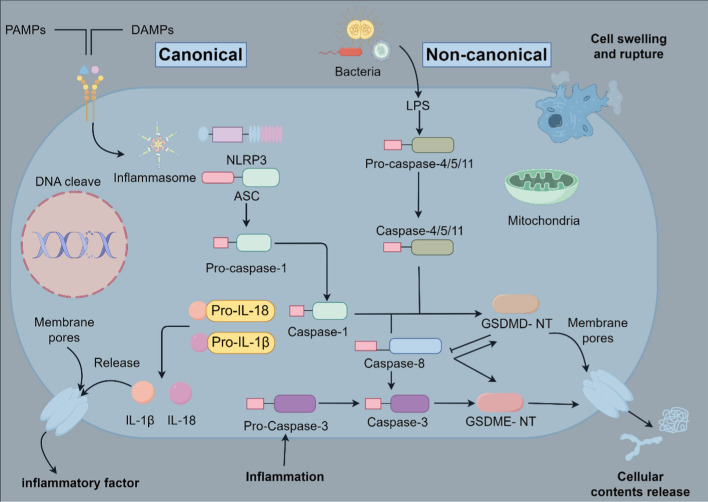
The function of caspase-8 in cellular pyroptosis. Beyond the canonical pyroptosis pathway orchestrated by caspase-1, as well as the noncanonical pathway facilitated by human caspase-4/5 and murine caspase-11, caspase-8 initiates an alternative pathway that operates independently of caspase-1 and caspase-4/5/11, targeting the same cleavage site on gasdermin D (GSDMD) as caspase-1. This GSDMD-dependent pyroptosis can suppress the activation of caspase-8 within the inflammasome, effectively halting the processes described above. Furthermore, caspase-8 can act as a precursor protein to activate caspase-3, which subsequently cleaves gasdermin E (GSDME) to modulate pyroptosis, or caspase-8 can directly cleave GSDMD or GSDME to trigger pyroptosis (by Figdraw)

In conclusion, the role of caspase-8 in pyroptosis extends beyond its conventional association with apoptosis, demonstrating a multifaceted function in the regulation of cell death pathways. Caspase-8 can initiate pyroptosis independently of caspase-1 and caspase-4/5/11, potentially acting as a supplementary mechanism in the defense against infections. Additionally, caspase-8’s capacity to scaffold inflammasome assembly and activate caspase-1 further highlights its critical role in inflammatory responses. This dual functionality establishes caspase-8 as a pivotal regulator in balancing apoptosis, necroptosis, and pyroptosis, underscoring its potential as a therapeutic target for inflammatory diseases and infections. The role of caspase-8 in pyroptosis is depicted in Fig. 3.

### Caspase-8 and PANoptosis

**Fig. 4:**
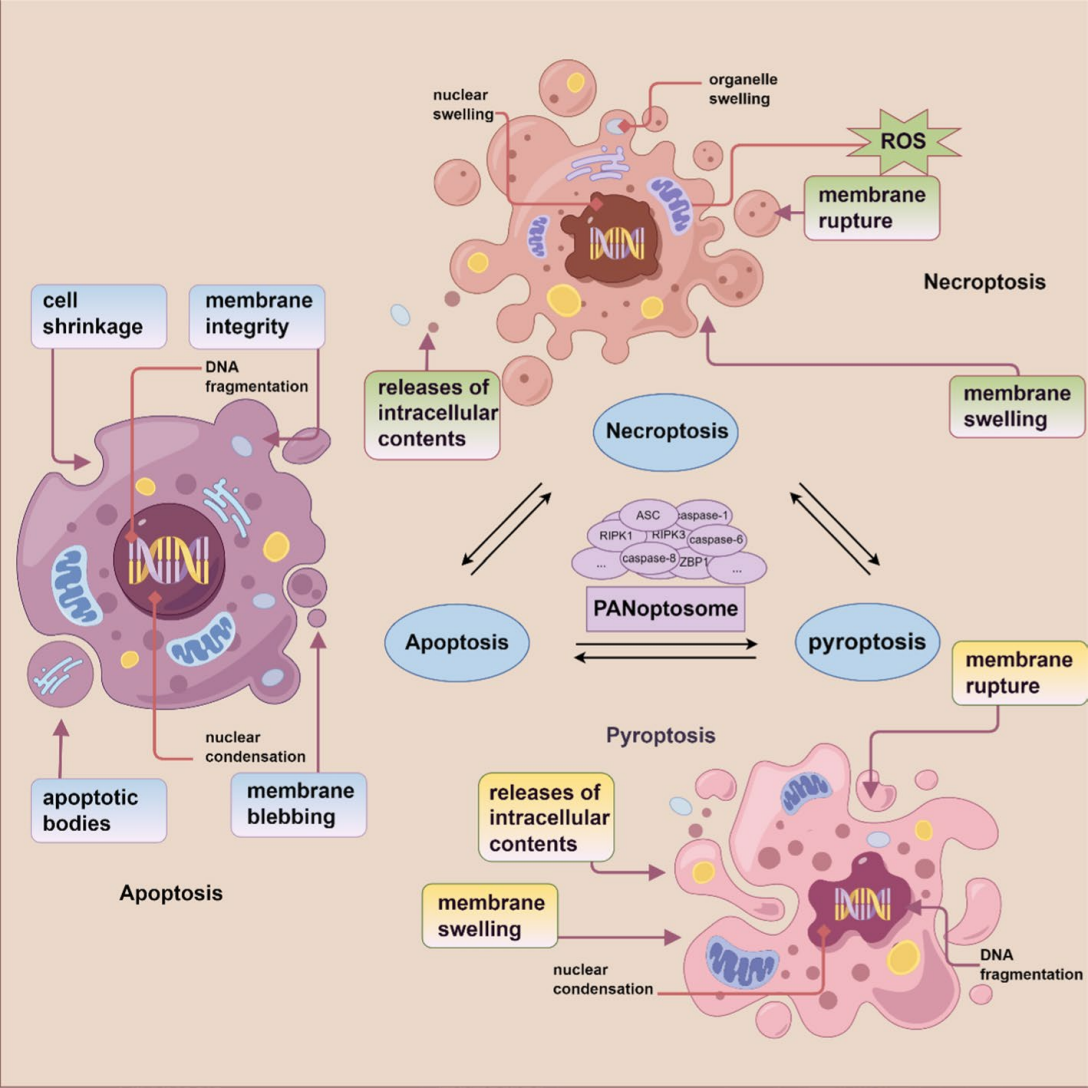
Schematic representation of PANoptosis and the associated triad of cell death pathways. PANoptosis embodies a comprehensive form of cell death that incorporates pyroptosis, apoptosis, and necroptosis. It is governed by a cytoplasmic protein conglomerate known as the PANoptosome, which encompasses core elements from various cell death modalities, including caspase-8. Within PANoptosis, the inhibition of one death pathway precipitates the activation of an alternative inflammatory cell death route. The PANoptosome serves as a conceptual scaffold, orchestrating the recruitment of pivotal molecules from disparate cell death pathways to facilitate the process of inflammatory cell death (by Figdraw)

Apoptosis, necroptosis, and pyroptosis are all manifestations of PCD, acting as an intrinsic protective mechanism orchestrated by the organism itself. Despite the potential harm on the organism, their roles in development and survival are crucial [78, 79]. Often, the functions of these cell death pathways overlap, making them difficult to differentiate. To address this complexity, scholars have proposed a new, comprehensive form of cell death that encompasses apoptosis, necroptosis, and pyroptosis, termed PANoptosis [77]. This newly defined cell death modality is regulated by a cytosolic protein complex known as the PANoptosome. The initial components identified in the PANoptosome include RIPK1, ASC, NLRP3, and caspase-8 [80], with later studies adding RIPK3, caspase-6, ZBP1, and caspase-1 to the list [81]. The structural domains conducive to homo-interaction, found in many proteins involved in PCD pathways—including CARD, DD, DED, PYD, and receptor-interacting protein homotypic interaction motif (RHIM)—along with protein–protein interactions, provide the molecular foundations for the assembly of the PANoptosome. Within the PANoptosome, all three forms of cell death—apoptosis, necroptosis, and pyroptosis—can be activated concurrently. Blocking one pathway can trigger another inflammatory cell death pathway. Caspase-8, the traditional initiator of apoptosis, plays a pivotal role in PANoptosis, acting as a key molecule that interlinks the three death modalities. Regulating caspase-8 can directly influence PANoptosis. For instance, caspase-8, apart from initiating apoptosis, can mediate pyroptosis, and its activity can directly inhibit MLKL-mediated necroptosis. Studies have shown that when pyroptosis is inhibited, caspase-8 can activate an inflammation-based cell death termed secondary pyroptosis or apoptosis, via the assembly of inflammasome mechanisms [70, 71, 73, 82–85]. These findings underscore that the activation of apoptosis, necroptosis, and pyroptosis during PANoptosis is regulated by a common complex. Therefore, within PANoptosis, the PANoptosome can act as a conceptual scaffold, recruiting core molecules from different cell death pathways to execute the inflammatory cell death process. Within this intricate framework, interacting proteins with diverse catalytic functions ensure the progression of cell death [85]. The assembly of the PANoptosome is flexible, allowing for the timely recruitment and functional deployment of core components from different cell death pathways. However, as a complex and programmed process, PANoptosis inevitably is influenced by external factors such as host factors and pathogen proteins, including the synthesis of related proteins and assembly of complexes, and the activity of various effectors. From the initiation of cell death signals to the triggering of cell death and the generation of multiple effects, numerous effector proteins and signaling pathways are involved. Thus, regulating the expression of these proteins and signaling pathways could be an effective approach to modulating PANoptosis [86]. Among these, caspase-8, serving as a molecular switch between the three types of cell death, undoubtedly draws significant attention from researchers and scholars. A schematic of PANoptosis and the related three cell death pathways is depicted in Fig. 4.

### Caspase-8 and inflammasome

Innate immunity serves as the primary defense against infections, playing a critical role in the detecting and recognizing pathogenic microorganisms and necrotic cells [87, 88]. Relying on PRRs, the innate immune system can identify PAMPs and DAMPs, thereby triggering the activation of subsequent anti-infection mechanisms [89]. However, with the discovery of more innate immune receptors, it become evident that not all innate immune receptors recognize danger signals through direct binding to PAMPs or DAMPs. Instead, many discern harmful conditions by detecting cellular disturbances exerted by PAMPs or DAMPs. In most cases, upon detecting these disturbances, the receptors induce the expression of cytokines, inflammatory mediators, and other factors that enhance the defense mechanisms of host cells. Nonetheless, the host can also inhibit pathogen dissemination by inducing cell death, a process that is generally beneficial to the host [14, 90]. Inflammasomes are cytosolic supramolecular complexes that activate caspase-1 or other inflammatory caspases [91–94], with some inflammasome sensors being PRRs themselves. As discussed, PRRs encompass various receptor families, with the NLR family being among the most extensively studied. The NLRC4 inflammasome, for instance, can activate caspase-8-mediated apoptosis through the interaction between SUG1 and FADD, while in epithelial cells, caspase-8 can be activated by the NLRP3-ASC complex formed in mitochondria, thereby influencing the apoptosis process it mediates [95, 96]. Inflammasomes play a crucial role in regulating caspase-8 activity. Specifically, in macrophages, caspase-8 can be activated by the NLRP3, AIM2, and NLRC4 inflammasomes, while in dendritic cells (DCs), it is activated primarily by the NLRP3 inflammasome. This activation mechanism involves the recruitment of caspase-8 to the inflammasome complex, where it undergoes conformational changes and cleavage, leading to its activation. This interplay between inflammasomes and caspase-8 highlights a multifaceted regulatory axis that influences both inflammatory responses and PCD. A novel perspective suggests that manipulating the inflammasome-caspase-8 axis could offer innovative therapeutic strategies for diseases characterized by aberrant cell death and inflammation [73, 97–101]. Future research should focus on elucidating the precise molecular dynamics of this interaction and exploring how selective modulation of these pathways can be leveraged to treat chronic inflammatory and autoimmune disorders.

Moreover, caspase-8 also plays a regulatory role with inflammasome activity, with its effect varying depending on the cell type [14]. In macrophages, for example, caspase-8 and FADD can promote the production of IL-1β by activating both canonical and noncanonical NLRP3 inflammasomes. Conversely, in DCs, caspase-8 may inhibit the activation of the NLRP3 inflammasome mediated by the RIPK1–RIPK3–MLKL pathway [102, 103]. Caspase-8 has also been implicated in an alternative NLRP3 activation process mediated by TLR4, exhibiting monocyte specificity and species specificity, as it occurs only in human and pig monocytes, not in mouse monocytes. In this context, NLRP3, as a relevant factor, can be activated by the adapter protein TIR domain-containing adapter molecule 1 (TICAM1, also known as TRIF) and caspase-8 [104, 105]. Furthermore, necroptotic cell death can also activate NLRP3; when caspase-8 activity is inhibited, necroptosis occurs, and RIPK3-mediated formation of MLKL pores on the plasma membrane leads to the efflux of potassium ions, thereby promoting the activation of NLRP3. It is worth noting that the activation of caspase-8 downstream of RIPK3-mediated TLR3, can also independently activate NLRP3, separate from MLKL activation, highlighting the intertwined role of RIPK3-mediated NLRP3 activation and pro-inflammatory potential of necroptosis [104, 106–108]. Studies also indicate that caspase-8 can participate in the regulation of upstream inflammasomes of NLRP3 [108, 109]. Thus, while the precise molecular mechanisms by which caspase-8 is involved in these processes are not fully elucidated, its role in the regulating the expression and maturation of inflammasomes and inflammatory mediators provides new insights into targeting caspase-8 for regulating cell death and maintaining cellular homeostasis.

In summary, the interaction between caspase-8 and inflammasomes constitutes a central regulatory axis for inflammatory responses and programmed cell death (PCD). Given these intricate dynamics, we propose that therapeutic strategies targeting the inflammasome–caspase-8 axis could effectively manage diseases characterized by dysregulated inflammation and cell death. Future research should aim to elucidate the precise molecular interactions governing this axis, potentially unveiling new, selective methods to modulate these pathways for therapeutic benefits in chronic inflammatory and autoimmune disorders. This approach not only provides a promising avenue for disease intervention but also deepens our understanding of the fundamental processes controlling immune homeostasis.

### Caspase-8 and inflammatory diseases

While the apoptotic function of caspase-8 has been extensively studied, its nonapoptotic roles have also attracted considerable attention, especially for their importance in cellular development [14]. Mice deficient in caspase-8 exhibit embryonic lethality at E10.5, and similarly, mice harboring catalytically inactive caspase-8 mutations, casp8^C382A/C362A^, also display embryonic lethality. Although this observation does not necessarily implicate a non-cell death function of caspase-8, it underscore the essential role of caspase-8 activity in embryonic development [102, 110]. In humans, aberrant expression of caspase-8 is associated with a range of refractory diseases, including autoimmune lymphoproliferative syndrome (ALPS), immunodeficiency, inflammatory bowel disease (IBD), neurodegenerative disorders (NDDs), and cancer. Research indicates that inhibiting the nonapoptotic functions of caspase-8 may help suppress excessive inflammatory gene transcription, including key inflammatory molecules such as NLRP3 and IL-1β, thereby alleviating inflammation. Enhancing the expression of caspase-8 could potentiate its apoptotic or necroptotic defenses against tumors or infections [10]. Therefore, understanding the relationship between caspase-8 and disease onset, as well as the potential molecular mechanisms or signaling pathways involved, is of paramount importance. It is essential to summarize and review the role of caspase-8 in relevant diseases to gain a deeper understanding of its function within disease contexts, thereby providing a reference and inspiration for the design and development of targeted therapeutics focusing on caspase-8 or related pathways in cell death.

### Caspase-8 and autoimmune diseases

Multiple sclerosis (MS) is a chronic, inflammatory, autoimmune demyelinating disease predominantly affecting the central nervous system (brain and spinal cord), characterized by multifocal demyelinating lesions [111]. The exposure to a plethora of pro-inflammatory agents within the meninges is a principal factor leading to pathologic alterations in the MS cortex. Postmortem studies of cortical gray matter from individuals with progressive MS have revealed an upregulation of TNFR1/RIPK1 signaling within cortical neurons. This upregulation triggers downstream necroptotic cell death via the RIPK1–RIPK3–MLKL pathway, contributing to the formation of necroptotic bodies within the MS cortex and playing a role in the pathological progression of MS. Concurrently, a downregulation of caspase-8 levels and caspase-8-dependent apoptotic signaling in the MS cortex inhibits apoptosis. This suggests that neuronal death in the MS cortex occurs through necroptosis rather than apoptosis, with the downregulation of caspase-8 possibly being a key reason for the shift from apoptotic to necroptotic cell death [112]. Ofengaim et al. also found a close association between caspase-8 deficiency, activation of necroptotic cell death, and MS. They observed defects in caspase-8 activation within MS cortical lesions, along with oligodendrocytes expressing RIPK1, RIPK3, and MLKL, indicating the activation of necroptosis. Given the critical role of caspase-8 in inhibiting necroptosis, these findings suggest that caspase-8 deficiency and the resultant disinhibition of necroptosis could contribute to MS progression [113]. Similarly, research has identified reduced expression levels of caspase-8 in MS patients with detectable gadolinium-enhancing lesions [114, 115]. Studies by Kim et al. also highlighted the negative regulatory role of caspase-8 in autoimmune diseases like MS within infiltrating macrophages. Caspase-8 can inhibit the production of IL-1β during the inflammatory demyelination process and autoinflammatory responses, with its deficiency commonly accompanied by inflammatory activation and inflammasome activation [111]. This suggests that elevated levels of caspase-8 might represent a potential strategy for suppressing autoimmune diseases, whereas reduced levels of caspase-8, leading to dysregulated inflammatory responses, could exacerbate the disease. However, contrary to oligodendrocytes, active caspase-8 level is significantly increased in microglia of the brain tissue of MS patients, contributing to the activation of noncanonical inflammasomes and the production of inflammatory cytokines/chemokines, thereby aggravating the disease [113, 116]. Driven by inflammatory responses, activated microglia release pro-inflammatory mediators, further exacerbating the progression of MS. This reveals the cell-specific and diverse functionality of caspase-8 across different cell types, highlighting its varying roles in apoptosis, necroptosis, pyroptosis, and inflammasome activation. These findings underscore the need for further investigation into the differential mechanisms of caspase-8 activation. It is conceivable that caspase-8, with its multifaceted roles in MS, could emerge as a significant target in the therapeutic intervention of MS progression.

IBD represents a chronic state of inflammation within the gastrointestinal tract, primarily comprising ulcerative colitis (UC) and Crohn’s Disease (CD). Numerous studies suggest that immune dysregulation towards the gut microbiota causes the immune system to mistakenly target the normal tissues of intestine, resulting in chronic inflammation and tissue damage [117–119]. While the exact etiology of IBD is not fully understood, it is often considered an autoimmune condition. Caspase-8 plays a significant role in cell death and inflammasome activation, among others, thereby influencing the progression of IBD from various aspects. Additionally, caspase-8 is involved in the homeostasis of intestinal immunity and inflammation. For instance, genetic caspase-8 deficiencies in patients have been associated with intestinal inflammation and immune dysregulation, accompanied by gastrointestinal symptoms such as diarrhea and perianal diseases [120]. Research by Robin et al. discovered that the regulating intestinal epithelial cell (IEC) death is crucial for maintaining the homeostasis of intestinal inflammation, and its imbalance could be one of the mechanisms underlying the development of IBD. Z-DNA binding protein 1 (ZBP1) and TNFR1 drive the inflammatory response in IECs with FADD deficiency through inducing MLKL-mediated necroptosis and caspase-8–GSDMD-mediated pyroptosis, whereas FADD and caspase-8 are able to regulate the intestinal inflammation and homeostasis through these pathways [121]. Furthermore, ZBP1 also plays a role in the intestinal inflammation of humans with caspase-8 mutations, potentially progressing to a severe form of IBD. Studies have shown that a deficiency in caspase-8 can elevate the activity of inflammasomes through the upregulation of RIP3, underscoring the close relationship between necroptosis and inflammatory tissue damage in IBD, with caspase-8 deficiency being a primary reason for the disinhibition of necroptosis [122]. In a case report by Kanderova et al., an early-onset IBD linked to caspase-8 mutation was discovered, and the direct reasons behind the clinical phenotype caused by the caspase-8 mutation were explored [123]. These studies suggest that reduced levels or defects in caspase-8 could provoke IBD. However, some studies present an entirely contrasting viewpoint. A study by Chen et al., which consolidated proteomics data and conducted a prospective Mendelian randomization (MR) analysis, found a causal relationship between elevated levels of caspase-8 and an increased risk of UC [124]. Additionally, a cross-sectional study by Moraes et al. indicated that elevated levels of caspase-8 might promote UC progression through interactions with inflammatory factors [125]. Research by Becker et al. showed an increase in caspase-8 expression in the colonic mucosa of rats induced with UC by acetic acid (AA), suggesting that caspase-8 plays a role in controlling the necrosis of Paneth cells related to mucosal inflammation and the potential death of IECs in patients with CD [11]. Therefore, targeting caspase-8 or its regulators could be a potential avenue for preventing IBD. However, despite extensive studies on the involvement of caspase-8 in cell death, research into its non-cell death functions, such as pro-inflammatory actions, remains relatively underexplored. Given the complex role of caspase-8 in the pathogenesis of IBD, further in-depth research is warranted.

ALPS represents a rare genetic disorder characterized by an autoimmune response due to excessive lymphocyte proliferation. A systematic review on ALPS revealed that approximately 85% of ALPS and ALPS-like cases are associated with mutations in the FAS gene, suggesting that mutations in the FAS gene could be a principal etiological factor [126]. Numerous studies have also indicated that lymphocyte apoptosis dysregulation mediated by FAS is a contributing factor to the disease [127–131]. However, some ALPS or ALPS-like patients exhibit normal FAS gene expression and sequencing, with apoptosis dysregulation occurring downstream of FAS, such as in patients with caspase-8 deficiency. Caspase-8, a pivotal apoptotic protease, plays an essential role in the FAS-mediated apoptosis pathway. The first discovery of two siblings with homozygous caspase-8 mutations was made in 2002, characterized by lymphadenopathy and defective lymphocyte apoptosis [12]. In the research conducted by Julie et al., two patients from the same extended family as the aforementioned individuals, bearing identical mutations, were identified. These patients presented symptoms akin to those with FAS mutation-associated ALPS, but with more pronounced immunodeficiency symptoms [132, 133]. Studies have suggested that caspase-8-associated apoptosis and necroptotic death collectively influence the progression of ALPS. The impairment of FADD–caspase-8-induced apoptosis, resulting from ablation of RIPK3 or MLKL, has been identified as a causative factor for ALPS [14]. Although it is certain that the disorder is closely linked to disruptions in FAS and caspase-8-related apoptotic pathways, the rarity of the disease and the limited number of cases have impeded in-depth research into the association between caspase-8 expression, deficiency, and ALPS.

Furthermore, numerous autoimmune disorders have been confirmed to be closely associated with caspase-8. Research has indicated that caspase-8 may play a role in the pathological progression of rheumatoid arthritis (RA), functioning within synovial antigen-presenting cells, and modulating the response to inflammatory stimuli through the inhibition of necroptotic death, thereby maintaining homeostasis within the joint [134]. The study by Zheng et al. also discovered that caspase-8, by regulating ferroptosis and pyroptosis, participates in the RA process and serves as a crucial biomarker for both ferroptosis and pyroptosis in RA, suggesting that targeting caspase-8 could be a potential therapeutic strategy for RA [135]. Moreover, in autoimmune hepatitis (AIH), excessive activation of apoptosis, especially caspase-8-mediated apoptosis, is the primary mechanism of cell death. This suggests that intervening caspase-8-mediated extrinsic apoptosis pathway could be a viable approach to protect liver cells in AIH [136]. Additionally, autoantibodies extracted from the serum of patients with Sjögren’s Syndrome (SS) can trigger a caspase-8 dependent apoptosis pathway, thereby mediating the death of A-253 human salivary gland cell lines, indicating an indispensable role of caspase-8 in the pathogenesis of SS [137]. In conclusion, caspase-8 is implicated in the pathological processes of various autoimmune diseases, offering new targets and perspectives for treatment. However, it is essential to note that the role of caspase-8 in these diseases may be complex, requiring further research to elucidate the specific mechanisms.

### Caspase-8 and neurodegenerative diseases

NDDs represent a diverse group of neurological disorders that are often age-associated and characterized by pathological alterations including apoptosis, necroptosis, oxytosis, and ferroptosis among others [138]. These conditions lead to progressive loss of neurons within the CNS or the peripheral nervous system (PNS), culminating in the collapse of neural networks and neuronal damage, ultimately resulting in impairments in memory, cognition, behavior, sensory, and/or motor functions [139, 140]. NDDs are marked by the gradual decline of neurological functions, presenting a significant challenge for caregiving and treatment while causing considerable suffering on affected individuals. Recent research has underscored the pivotal role of caspase-8 as a key regulator for apoptosis, pyroptosis, and necroptosis, and its unique contribution to the pathogenesis of NDDs such as Alzheimer’s disease (AD), Parkinson’s disease (PD), and MS. Our previous discussions have delved into the potential mechanisms and roles of caspase-8 in MS. Herein, we focus on the other two most prevalent NDDs, AD and PD.

Globally, approximately 44 million individuals are affected by AD, which is characterized by cerebral atrophy and potential neuronal loss, with dementia being the most common manifestation, leading to progressive memory loss and cognitive impairments [141–146]. As the disease progresses, patients often require continuous care and assistance, imposing a significant burden on families and society [147]. Amyloid-beta (Aβ) and tau protein, particularly its hyperphosphorylated form (ptau), are critical in disease development. The accumulation of extracellular plaques formed by Aβ and neurofibrillary tangles (NFTs) within neurons are key neuropathological hallmarks of AD [141, 146, 148]. Recent studies suggest the caspase family may be involved in the formation of plaques and NFTs, with caspases capable of cleaving tau, thereby initiating or exacerbating the formation of tau tangles [149]. Caspase-8 has been identified as playing a significant role in the progression of AD, acting upon amyloid precursor protein induced by Aβ1–40 to instigate apoptosis [150]. Research indicates that caspase-8 can cleave amyloid precursor protein (APP) during apoptosis, leading to an increased Aβ formation and thus contributing to the onset of AD [151]. Furthermore, caspase-8 has been found to promote neuronal apoptosis and AD-related motor dysfunction, suggesting that inhibiting caspase-8 could be a promising avenue for slowing AD progression [152]. Additionally, brain immune cells such as astrocytes and microglia, which are involved in AD progression, release pro-inflammatory cytokines including IL-1β, TNF-α, and IL-6, thus promoting tau hyperphosphorylation. The activation of the immune system can induce cell death through various mechanisms including pyroptosis, apoptosis, and necroptosis, potentially leading to the release of pro-inflammatory cytokines and the occurrence of chronic neuroinflammation, exacerbating the severity of AD [147]. Caccamo et al. observed a significant presence of necroptosis in the brains of individuals posthumously diagnosed with AD, and in AD mouse models, inhibiting necroptosis was found to reduce cell death [153]. Furthermore, inflammasomes and their mediated processes of inflammation and pyroptosis also constitute pathological factors in NDDs including AD, where inflammasomes such as NLRP1, NLRP3, and AIM2 play a pivotal role in the proliferation of Aβ pathology [154]. Caspase-8 emerges as a critical regulator of these cell death processes and neuroinflammatory mediators, playing a significant role in the onset and progression of AD. However, its function within the pathogenesis of AD remains underexplored. Recent research has shown that in AD mouse models, the caspase-8/RIPK3 axis is essential for promoting Aβ deposition and gliosis, which are indispensable for the progression of AD. Additionally, it was confirmed that the combined deficiency of caspase-8 and RIPK3 could limit the activation of the NLRP3 inflammasome and the secretion of IL-1β, thereby curbing the pathological progression of AD. Consequently, caspase-8, as a crucial regulator driving the expression of inflammasome genes and the release of IL-1β in response to Aβ, is vital to the development and progression of AD [155]. Despite these insights, the role of caspase-8 in AD is still in its early stages of exploration. This suggests that caspase-8 might also control other aspects of AD progression, with its regulatory impact on AD pathology harboring much undiscovered potential. Targeting caspase-8 could represent a novel strategy for limiting AD and neuroinflammation, making its specific role in AD an uncharted territory ripe for exploration.

PD, alongside AD, ranks among the most prevalent NDDs in the elderly, marked by the progressive loss of dopaminergic neurons in the substantia nigra, accompanied by neurodegeneration within the substantia nigra pars compacta [156–158]. Similar to other NDDs, the etiology of PD is highly complex, likely involving oxidative stress, neuroinflammation, mitochondrial dysfunction, and neuronal death, with its fundamental cause remaining elusive [156]. The involvement of caspase-8 in PD is multifaceted, potentially influencing the progression of disease through its regulation of apoptosis, necroptosis, and inflammatory responses, making it a valuable target for PD treatment research. Apoptosis is considered the principal mechanism of neuronal death in most NDDs, including PD. This apoptotic process has been extensively documented in PD patients and various experimental models, such as MPTP mouse models, with apoptosis capable of being reversed through caspase inhibition [159]. In patients with PD, apoptotic-like changes in the affected cells of substantia nigra are significantly more prominent than in healthy controls, suggesting that apoptosis may play an essential role in the pathological progression of PD [160–162]. Caspase-8, as one of the initiators of apoptosis, promotes cell death by cleaving various substrates and may significantly contribute to the chronic inflammation observed in PD, which can persist for decades [159]. Beyond apoptotic factors, necroptosis has also been implicated in the pathogenesis of PD. Clinical studies have reported a marked increase in degenerative changes in dopaminergic neurons within the substantia nigra of PD patients, with a significant elevation in necroptosis markers RIPK1, RIPK3, and MLKL compared with control groups [163]. The absence of RIPK3 has been shown to protect against neurodegeneration in dopaminergic neurons, supporting the hypothesis that necroptosis may be one of the causes for neuronal cell death in PD [156]. The heterodimer of procaspase-8 and cFLIP_L_ exhibits proteolytic activity, mediating the cleavage of RIPK1 and inhibiting necroptosis without inducing apoptosis under physiological conditions. Hence, activated caspase-8 plays a pivotal role in suppressing RIPK1-mediated necroptosis [60, 159]. Furthermore, compelling evidence suggests that inflammatory factors significantly influence cell death in PD progression [164–166]. Pro-inflammatory microglial activation may be a critical element in the progression of chronic neuroinflammation associated with PD. The resulting inflammatory mediators can persist longer than normal, potentially leading to caspase-dependent neuron death (including caspase-8/3/7 pathways), regulating microglial activation and associated neurotoxicity [167, 168]. In MPTP mouse models of PD, researchers found that the activation of pro-inflammatory microglia could disrupt the dopaminergic system of the nigrostriatal pathway, and the absence of caspase-8 could block microglial activation in the MPTP model of PD, suggesting that caspase-8 may play a pro-inflammatory role in the pathology of PD [167]. Additionally, research has discovered that caspase-8 can regulate in early-onset familial PD with autosomal recessive mutations in the F-box only protein 7 gene (Fbxo7). Caspase-8 can interact with FBXO7, triggering its activation and leading to the degradation of the cell-protective factor FOXO4, a process that could weaken neuronal protection and accelerate cell death [169]. These findings indicate that caspase-8 may play an essential role in neuronal death and holds a crucial position in the etiology of PD, potentially serving as a promising therapeutic target. However, most research to date has primarily been conducted on experimental models, and the regulatory role of caspase-8 in PD through various pathways is complex, making ti difficult to consolidate into a single model. Consequently, a unified consensus on the therapeutic effects of caspase-8 has yet to be established. This area of research remains in its preliminary phases, calling for further in-depth studies into the molecular mechanisms involved.

### Caspase-8 and sepsis

Sepsis, characterized by a dysregulated host response to infection and complex organ dysfunction, is a prevalent complication among critically ill patients. It is associated with a high morbidity rate and can cause lethal damage to various organs [170–172]. Presently, sepsis is a leading cause of mortality worldwide in noncardiac intensive care units, with death rates soaring between 20 and 25% [173]. Prior research has highlighted the intricate pathophysiology of sepsis and the lack of effective treatment modalities, underscoring the importance of identifying novel therapeutic targets for its management [174]. The mechanism of sepsis involves an imbalance in the host's innate and adaptive immune responses. In the early stages, this imbalance is often accompanied by an overactive immune system and inflammatory response, alongside immunosuppression. Numerous immune cells are activated, regulating immune and inflammatory processes and accelerating the progression of sepsis [175]. At the cellular level, various forms of cell death, including apoptosis, necroptosis, necrosis, autophagy, and ferroptosis, contribute to the advancement of sepsis [175, 176].

Caspase-8 is implicated in numerous aspects of the septic pathology and emerges as a potential therapeutic target. Studies have shown significant differences in caspase-8 levels between sepsis survivors and nonsurvivors, with elevated blood caspase-8 levels in nonsurvivors correlating with higher mortality rates [177]. Moreover, the caspase-8-mediated apoptotic pathway may play a pivotal role in the progression of sepsis. Within renal endothelial cells, tumor necrosis factor TNF-α receptor 1 induces apoptosis through a caspase-8-dependent extrinsic apoptotic pathway, potentially exacerbating sepsis-induced acute kidney injury (SI-AKI) [178–181]. Annexin A1 (ANXA1) has been shown to mitigate inflammation and apoptosis in vitro and in vivo through an Fpr2 receptor-dependent pathway, thereby alleviating sepsis-induced SI-AKI [182]. Nevertheless, not all pro-apoptotic processes contribute to the progression of sepsis. Studies indicate a positive correlation between the severity of sepsis and the levels of anti-apoptotic activity in neutrophils; when apoptosis in neutrophils is significantly inhibited, the delayed apoptosis may lead to an intensified inflammatory response and multiple organ dysfunction in septic patients, indicating that the level of neutrophil apoptosis may serve as an assessment indicator for the severity of sepsis [176, 183–185]. In this context, dysregulation of caspase-8 phosphorylation or inhibition of its catalytic activity can contribute to the survival of sepsis-induced neutrophils by inhibiting apoptotic pathways [186, 187]. For instance, the adenosine 2A receptor (A2AR) can inhibit neutrophil apoptosis by blocking the signaling pathways of caspase-8, caspase-3, and polyadenosine diphosphate ribose polymerase (PARP) by suppressing autophagy [188]. Thus, caspase-8 mediated inhibition of apoptosis may represent a potential strategy for treating and assessing the level of sepsis. In addition to the apoptosis, pyroptosis has been demonstrated to contribute to the regulation of sepsis. Although pyroptosis was initially perceived as detrimental to the host, moderate levels of pyroptosis can serve as a mechanism for pathogen elimination, thereby facilitating self-protection. Specifically, in the context of caspase-8-mediated pyroptosis, caspase-8 is capable of cleaving gasdermin D (GSDMD) and gasdermin E (GSDME), resulting in the formation of “gasdermin pores” in the cell membrane. These pores mediate pyroptosis and subsequently trigger inflammatory responses [75, 76, 102, 189]. Research demonstrates that activated GSDMD, by forming “gasdermin channels” in the bacterial cell membrane, can kill *Escherichia coli*, *Staphylococcus aureus*, and *Listeria monocytogenes*, thereby offering some protection against sepsis [190]. Pyroptosis appears to have a dual role in the pathogenesis of sepsis: a moderated level of pyroptosis could offer protective effects, whereas excessive pyroptosis might trigger severe inflammatory responses, accelerating the progression of sepsis [102, 189–191]. In recent years, necroptosis has also been identified as playing a pivotal role in the pathophysiology of sepsis. However, whether cells undergo apoptosis or necroptosis may depend on the activity of caspase-8. Although the precise molecular mechanisms remain unclear, this suggests that caspase-8 activity might influence sepsis progression by regulating necroptosis. Necroptosis has been confirmed to participate in the processes of sepsis and its complications, where inhibiting necroptosis could alleviate lung [192], kidney [193], and liver [194] damage caused by sepsis. Studies have shown that the absence of RIPK3 can confer protective effects in sepsis models, and inhibiting RIPK3 or RIPK1 can also reduce organ dysfunction and inflammatory responses in septic mice, highlighting the potential of RIPK kinase inhibitors in sepsis-related treatments [192, 195–197]. Further research has discovered that protein tyrosine phosphatase nonreceptor type 6 (PTPN6) can target the RIPK1–RIPK3–MLKL pathway and the caspase-8-mediated apoptosis pathway, thereby inhibiting platelet apoptosis and necroptosis during sepsis and preserving platelets from death, which helps maintain vascular integrity during sepsis [198]. Additionally, RIPK3-mediated necroptosis and GSDMD-induced pyroptosis have been proven to act synergistically during sepsis, intensifying inflammatory signaling pathways and tissue damage, thus exacerbating the progression of sepsis [199].

It can be concluded that multiple cell death pathways are involved in the progression of sepsis, highlighting the complex role of caspase-8 in sepsis. The overlapping roles of pyroptosis, apoptosis, and necroptosis in cell death make it challenging to fully distinguish between these processes [200]. For instance, while neutrophil apoptosis is inhibited during sepsis, caspase-8 activity is likely suppressed as well, but the specific role and molecular mechanism of neutrophil necroptosis remain unclear [187]. The concept of PANoptosis may provide further insight into these overlapping phenomena, as it suggests that the mechanisms of pyroptosis, apoptosis, and necroptosis are interconnected. This underscores that the role of caspase-8 in sepsis extends beyond a single cell death process or signaling pathway. Given its potent regulatory role in the cell death, a deeper investigation into caspase-8 is highly warranted, as many of its functions are still undiscovered.

### Caspase-8 and cancer

Caspase-8 functions as a critical regulator of cellular fate, with its importance in human development and homeostasis well established. Due to this pivotal role, caspase-8 has garnered considerable attention from researchers, especially regarding its function. Cancer, characterized by uncontrolled cell proliferation and disrupted cell cycle regulation, involves complex etiology, high genetic susceptibility, and challenging treatment resistance, making its therapy a formidable global medical challenge. Consequently, researchers continue to explore molecular pathways that could inhibit cancer progression [9, 201]. For a long time, it was believed that diminished expression/activity of caspase-8 facilitated apoptosis evasion, thereby reinforcing cellular resistance to radiotherapy and chemotherapy in most cancers. However, recent studies suggest that this may only characterize a minority of cancer types, with caspase-8 activity not universally diminished; in fact, it remains unchanged or even elevated in certain cancers, providing a proliferative advantage through its nonapoptotic functions [6, 202].

In cancers with reduced caspase-8 expression, such as hepatocellular carcinoma [203–205], breast cancer [206, 207], and Ewing's sarcoma [208, 209], the downregulation of caspase-8 activity occurs through genetic alterations (such as somatic, missense, and frameshift mutations, allelic losses or deletions) and post-translational modifications (PTMs, such as phosphorylation [210], ubiquitination [211], and nitrosylation [212]). This modulation mediates apoptosis suppression, allowing tumor cells to evade cell death. Additionally, PTMs of caspase-8 can promote cancer progression by enhancing cellular motility, migration, inflammation, angiogenesis, and resistance to genotoxic stresses [202, 213–216]. In summary, the oncogenic potential stemming from the diminished expression and activity of caspase-8 is substantial and aligns with its classical apoptotic functions.

In cases where caspase-8 expression remains relatively stable, such as in head and neck squamous carcinoma [217, 218], esophageal cancer [219, 220], and rhabdomyosarcoma [221, 222], caspase-8 activity may be compromised, often resulting in inhibited apoptosis. The underlying mechanisms may include dysfunctional procaspase-8 [223, 224], activation of anti-apoptotic NF-kB by caspase-8 mutants [217], and suppression by overexpression of Bcl and IAP family proteins [225, 226]. Moreover, tumor cells might also acquire enhanced migratory and invasive capabilities due to altered caspase-8 function [218, 227]. These processes collectively underscore that the most discernible impact of reducing caspase-8 expression or activity is the ability of cancer cells to evade caspase-8-mediated apoptosis. Simultaneously, the reduction in tumor cell death and the rapid cell proliferation are associated with an increased propensity for metastasis [6, 228].

Beyond the previously mentioned scenarios of diminished caspase-8 expression, the overexpression of caspase-8 in tumor cells—such as in colorectal cancer [229, 230], cervical cancer [231, 232], and renal cell carcinoma [233, 234]—also furnishes many tumor cells with a growth advantage. The most probable mechanism behind this phenomenon is the nonapoptotic functions of caspase-8 in the progression of these cancers. For instance, in glioblastoma multiforme (GBM), caspase-8 is co-opted by tumor cells and, paradoxically, exerts a pro-tumorigenic role. During this process, both the expression of caspase-8 and the activity of Src tyrosine kinase—which facilitates the phosphorylation of caspase-8 on tyrosine 380—are aberrantly activated. Although Src-mediated phosphorylation can inhibit caspase-8 activity, Contadini et al. [216] uncovered that Src-dependent phosphorylation of caspase-8 is a prerequisite for the activation of NF-κB, and can sustain and promote inflammation, angiogenesis, and resistance to radiotherapy [216, 235]. Additionally, throughout tumor development, the capacity of caspase-8 to promote cell motility [236], angiogenesis [235], and tumorigenic transformation [210] may provide substantial support for the initial tumor growth. However, as the cancer becomes more established, these functions might become redundant, and with the progression of malignancy, caspase-8 could be downregulated and play a role in promoting metastasis. This suggests that tumor cells might regulate the expression of caspase-8 by sensing changes in their growth microenvironment to control cell fate [6, 228, 237]. Mandal et al. [6] provided a comprehensive review of the variations of caspase-8 in different cancer types and the potential effects. Furthermore, they also discussed the genetic alterations in caspase-8 across various cancer types, such as promoter methylation, frameshift mutations, and missense mutations.

Past studies primarily focused on the apoptotic function of caspase-8; however, its nonapoptotic roles in tumors have recently emerged as a key area of research. Beyond its role in PCD, caspase-8 also participates in processes such as cytoskeletal remodeling, cell adhesion, and cell migration. Caspase-8 can act as part of various biological sensing complexes, exerting either pro-migratory or pro-death functions depending on the cellular microenvironment [214]. This nonapoptotic functionality does not rely on the enzymatic activity of caspase-8 but on its capacity to serve as a scaffold or adapter within specific protein complexes. Through this scaffolding function, caspase-8 facilitates cytoskeletal reorganization [238], focal adhesion turnover, and integrin recycling, thereby sustaining the migratory ability of apoptosis-resistant tumor cells both in vitro and in vivo [237, 239]. Moreover, the role of caspase-8 in inflammatory and immune system functions cannot be overlooked. It was initially discovered that caspase-8 could activate NF-κB independently of its catalytic activity [240]. Upon inflammatory stimuli, caspase-8 can promote NF-κB activation and cytokine release, likely achieved through its scaffolding functions [241–243]. However, the mechanisms by which caspase-8 regulates NF-κB are complex and may involve various molecules including but not limited to cFLIP and RIPK1, which also impact the modulation of NF-κB [14]. In addition, the regulatory effect of caspase-8 on various inflammasomes and pro-inflammatory cytokines also confers a pro-inflammatory role within the tumor microenvironment (TME) [216]. Understandably, given the variable expression of caspase-8 across different tumor types, its pro-inflammatory functions may also vary [202]. Caspase-8 is also a central protein in numerous signaling pathways, playing a significant role in the formation and maintenance of the TME. Beyond its substantial role in primary tumor cells, caspase-8 also exerts a pivotal influence within the TME by modulating immune responses, B-lymphocyte and T-lymphocyte activation, macrophage differentiation and polarization [244]. As outlined above, a growing body of evidence underscores the nonapoptotic roles of caspase-8 in cancer, with continuous discoveries highlighting its functions. These roles include but are not limited to acting as an enhancer of cell movement and migration [210, 214, 237], a promoter of tumorigenesis [235], a regulator of the cell cycle [202], a stabilizer of immune cell homeostasis and cytokine production [244], a scaffolding for specific protein complexes, and a promoter of inflammatory responses and angiogenesis to sustain the tumor growth environment [216].

Apoptosis is the primary signaling pathway through which tumor cells undergo cell death in the presence of effective drugs. However, tumor cells can evade death signals and counteract the effects of chemotherapeutic agents by manipulating the apoptotic process, thereby achieving a form of “immortality” [245]. Resistance mechanisms in tumor cells are facilitated by the downregulation of caspase-8 activity, with potential strategies including the reduced expression of procaspase-8, overexpression of inhibitors such as FLIP, and isolation by Bcl-2 (a protein localized in the mitochondrial membrane that inhibits cytochrome c release and downregulates apoptosis) [245–247]. For instance, in neuroblastoma (NB), caspase-8 is inactivated in about one-third of cases, and tissues with high malignancy and MYCN amplification often lack caspase-8 mRNA expression (248). Targeting caspase-8 could also be crucial for reducing the formation of metastatic lesions in neuroblastoma [249]. Moreover, primitive neuroectodermal brain tumor cells can also develop resistance to TRAIL-induced apoptosis through the loss of caspase-8 expression [250]. Similarly, overexpression of caspase inhibitors like cFLIP can downregulate apoptosis, as previously mentioned by blocking procaspase-8 from entering the DISC complex, inhibiting the activation of caspase-8. FLIP has been reported to inhibit apoptosis in various solid tumors, including melanoma and advanced Kaposi's sarcoma [245, 251–254]. Given the downregulation of caspase-8 in certain tumors, inducing caspase-8 expression in tumors that still retain it could therapeutically enhance the apoptotic sensitivity of tumor cells and suppress cancer growth [245]. Consequently, as caspase-8 is central to drug-induced apoptosis, it is a critical factor in tumor chemoresistance and may serve as a promising target in cancer therapy.

As delineated above, caspase-8 exhibits a pronounced dichotomy in its role within cancer therapies, with both its oncogenic and tumor-suppressive functions being substantiated or reported. In recent years, as research into cell death mechanisms has deepened, the apoptotic function of caspase-8 remains the most classical; however, its association with pyroptosis and necroptosis has also been affirmed to play roles in tumor progression. Studies have indicated that PD-L1 can transform TNFα-induced apoptosis into pyroptosis, mediating an atypical pathway of pyroptotic cancer cell death through the caspase-8/GSDMC pathway, resulting in tumor cell death [255]. Furthermore, research has discovered that caspase-8-mediated pyroptosis has implications for respiratory diseases, such as lung cancer, and may serve as a therapeutic target for related diseases [98]. Necroptosis has also been proven to proceed in a caspase-independent manner as a form of “cellular suicide” under the presence of caspase inhibitors, thereby limiting tumor growth, which suggests that apoptosis is not always the preferred mode of tumor cell death [9]. However, in many instances, distinguishing between pyroptosis, apoptosis, and necroptosis is challenging. PANoptosis, a more encompassing form of cell death, offers a better explanation of mechanisms in certain cancer progressions. In PANoptosis, a substantial release of inflammatory factors contributes to the formation of an inflammatory microenvironment. By affecting the dynamic equilibrium between cell death and regeneration, inflammation, and immune responses, PANoptosis can facilitate the transformation of normal cells into cancerous ones [256]. PANoptosis opens new avenues for the treatment of infectious, spontaneous diseases, and cancer, emphasizing the cross-talk between different cell death pathways, which plays a crucial role in tumor development. As a key protein intertwining pyroptosis, apoptosis, and necroptosis, caspase-8-mediated PANoptosis has significant implications in the mechanisms and progression of breast cancer, hepatocellular carcinoma, nasopharyngeal carcinoma, among others. This provides a robust foundation for cancer therapy. We anticipate that caspase-8 and its associated PANoptosis will be future research focus in the field of cancer treatment [9]. The relationship between caspase-8, certain diseases, and the associated cell death pathways as detailed in Table 1.

**Table 1:** Caspase-8 in diseases and associated cell death pathways

Disease name	The role of caspase-8 in diseases	Cell death pathway involved	References
Multiple sclerosis	Caspase-8 exhibits a complex role in MS, with its downregulation promoting necroptotic cell death in cortical neurons and oligodendrocytes, while its upregulation in microglia contributes to inflammation through noncanonical inflammasome activation, highlighting the need for further exploration of its differential effects across cell types	Apoptosis, necroptosis, pyroptosis, and inflammasome activation	[111–116]
Inflammatory bowel disease	Caspase-8 plays a dual role in IBD, with both its deficiency and overexpression linked to the disease's progression, highlighting its complex involvement in intestinal inflammation and homeostasis	Apoptosis, necroptosis, pyroptosis, and inflammasome activation	[11, 120–125]
Autoimmune lymphoproliferative syndrome	Caspase-8, a crucial mediator of apoptosis in the FAS pathway, is implicated in ALPS through its deficiency, which leads to dysregulated lymphocyte apoptosis and contributes to the disease’s progression, alongside potential involvement in necroptotic cell death	Apoptosis and necroptosis	[12, 14, 126–133]
Rheumatoid arthritis	Caspase-8 plays a multifaceted role in RA by modulating inflammatory responses to maintain joint homeostasis, serving as a pivotal biomarker and mediator for ferroptosis and pyroptosis, and enabling the resveratrol-induced apoptosis of fibroblast-like synoviocytes	Necroptosis, ferroptosis, pyroptosis, and apoptosis	[134, 135, 319]
Autoimmune hepatitis	In AIH, caspase-8 is the primary mediator of cell death through excessive activation of apoptosis, indicating that targeting the caspase-8-mediated extrinsic apoptosis pathway could be a potential therapeutic strategy to protect liver cells,	Apoptosis	[136]
Sjögren’s syndrome	In SS, caspase-8 is a pivotal mediator of cell death, both through autoantibody-triggered caspase-8 dependent apoptosis and via lysosome-dependent cell death caused by impaired autophagic caspase-8 degradation due to LAMP3 overexpression, underscoring apoptosis and lysosome-dependent pathways as critical mechanisms in the disease’s pathogenesis and potential therapeutic targets	Apoptosis	[137, 320]
Alzheimer’s disease	Caspase-8 plays a central role in AD by mediating neuronal apoptosis and contributing to the formation of amyloid plaques and neurofibrillary tangles, with its inhibition offering a potential strategy to slow AD progression	Apoptosis, necroptosis, pyroptosis, and inflammasome activation	[141, 146–155]
Parkinson’s disease	Caspase-8 plays a critical role in PD by regulating apoptosis, necroptosis, and inflammatory responses, contributing to the progressive loss of dopaminergic neurons and neurodegeneration, making it a potential therapeutic target for PD treatment	Apoptosis, necroptosis, and inflammatory responses	[60, 159–169]
Sepsis	Caspase-8 plays a critical role in sepsis by influencing cell death pathways, with elevated levels linked to mortality, and its modulation could serve as a potential therapeutic strategy for managing sepsis and its associated organ dysfunction	Apoptosis, necroptosis, pyroptosis, and inflammasome activation	[60, 176–188]
Hepatocellular carcinoma	Caspase-8, a pivotal regulator in both apoptosis and necroptosis pathways, is intricately involved in the modulation of HCC cell death, suggesting that the manipulation of caspase-8 activity could be a strategic approach for selectively targeting HCC cells through distinct cell death mechanisms	Apoptosis and necroptosis	[203–205, 321–324]
Breast cancer	Caspase-8 plays a pivotal role in breast cancer by mediating both extrinsic apoptosis and a noncanonical pyroptosis pathway, where it cleaves GSDMC to induce pyroptosis and tumor necrosis upon TNFα stimulation, and collaborates with PARP inhibitors to sensitize tumor cells to pyroptosis, thereby enhancing cancer cell death and immune response, highlighting its critical involvement in multiple cell death mechanisms in breast cancer	Apoptosis, necroptosis, and pyroptosis	[206, 207, 255, 325, 326]
Ewing’s sarcoma	Caspase-8 is a crucial mediator in the apoptotic pathways of Ewing’s sarcoma, as it regulates cell survival and proliferation through both extrinsic (Fas and caspase-8) and intrinsic (caspase-9, Bad, Bcl-2, and XIAP) pathways, and serves as a predictive biomarker for sensitivity to death receptor targeted agents like conatumumab, as well as playing a significant role in tumor cell death induced by TRAIL-expressing mesenchymal stem cells	Apoptosis	[208, 209, 327, 328]
Squamous carcinoma	Caspase-8 plays a pivotal role in the induction of apoptosis in squamous carcinoma cells, particularly in oral squamous cell carcinoma (OSCC), where it is activated by CLEFMA treatment and arsenic compounds, leading to both extrinsic and intrinsic apoptotic pathways, as evidenced by increased cleavage of poly ADP-ribose polymerase and activation of caspase-8, -9, and -3, ultimately contributing to the suppression of tumor growth	Apoptosis	[217, 218, 329, 330]
Non-small cell lung carcinoma (NSCLC)	Caspase-8 plays a critical role in non-small cell lung carcinoma (NSCLC) by regulating interleukin-8 production, being targeted for activation by HDAC inhibitors to induce apoptosis, and its overexpression along with FLIP correlates with poor prognosis; it is integral to both extrinsic and intrinsic apoptotic pathways in NSCLC	Apoptosis	[331–333]
Esophageal cancer	Caspase-8 plays a significant role in esophageal cancer by being involved in the induction of pyroptosis through the PKM2/caspase-8/caspase-3/GSDME axis following photodynamic therapy, while also being bypassed in TRAIL-induced apoptosis resistance mechanisms involving TRADD and c-FLIP, highlighting its involvement in both pyroptosis and apoptosis pathways in this cancer type	Apoptosis and pyroptosis	[219, 220, 334–336]
Colorectal cancer	Caspase-8 is a key mediator in colorectal cancer, as it is involved in the induction of apoptosis through the upregulation by sanshools and the sensitization effect of EGCG in combination with TRAIL, while also participating in the NAIP-NLRC4 inflammasome-mediated pyroptosis and inflammation	Apoptosis and pyroptosis	[229, 230, 337–340]
Renal cell carcinoma	Caspase-8 plays a critical role in renal cell carcinoma by mediating both apoptosis and pyroptosis, as evidenced by its activation in response to STING depletion, its involvement in TRAIL-induced apoptosis when combined with resveratrol, and its inhibition by miR-381-3p, which also suppresses necroptosis. Additionally, PP5 inhibition leads to the activation of caspase-8 in the extrinsic apoptotic pathway, suggesting its potential as a therapeutic target in RCC	Apoptosis and pyroptosis	[233, 234, 341–344]
Rhabdomyosarcoma	In rhabdomyosarcoma, caspase-8 plays a pivotal role in TRAIL-induced apoptosis, as its expression and catalytic activity are both necessary and sufficient for sensitivity to the DR5 antibody drozitumab. This sensitivity leads to the assembly of the death-inducing signaling complex and subsequent activation of the apoptotic pathway, resulting in cell death	Apoptosis	[221, 222, 327, 345, 346]
Ovarian cancer	Caspase-8 is a pivotal protease in ovarian cancer that orchestrates a delicate balance between apoptotic and nonapoptotic functions, such as cell cycle regulation, invasiveness, metastatic behavior, immune homeostasis, and cytokine production, with its dysregulation leading to increased tumor aggressiveness and immune resistance	Apoptosis	[244, 347, 348]
Nasopharyngeal carcinoma	Caspase-8 plays a crucial role in the anti-nasopharyngeal carcinoma action of calycosin, as it is identified as a core target in the network pharmacology analysis and validated in human and preclinical studies, with its activation leading to increased apoptosis in nasopharyngeal carcinoma cells	Apoptosis	[349–351]

### Regulation of caspase-8

Caspase-8 functions as a molecular toggle that determines cellular fate, with its regulation potentially impacting the pathogenesis of numerous diseases [29, 257]. Epigenetic modifications, which involve alterations in gene expression without changes to the DNA sequence itself [258], have been demonstrated to play a significant role. Studies in a mouse model of hepatocellular carcinoma have shown that genomic deletions at the caspase-8 locus do not silence caspase-8. Instead, significant hypermethylation of cytosine phosphate guanine (CpG) sites within the promoter sequence can lead to a deficiency in caspase-8 messenger RNA expression. This is attributed to the inactivation of a crucial promoter element spanning approximately 30 base pairs, which includes an SP1 binding motif colocated with CpG sites [259]. Moreover, Teng et al. has revealed that the methylation status of the caspase-8 gene promoter is associated with mRNA levels of caspase-8, kinase activity, and the anti-apoptotic and drug resistance capabilities of tumor cells in human malignant gliomas. This relationship suggests that caspase-8 methylation status could serve as a potential early diagnostic marker for these tumors [260]. These findings suggest that DNA methylation can influence the regulation of caspase-8 and may represent a critical target for future therapeutic strategies.

It is noteworthy that PTMs constitute an essential mechanism in the regulation of caspase-8. The types of PTMs affecting caspase-8 include phosphorylation [239], ubiquitination [261], and S-nitrosylation [262], among others. Caspase-8 can determine cell fate either by proteolytic cleavage of target proteins or by recruiting them into multi-protein complexes, with proteolysis being a strictly regulated irreversible process. Consequently, reversible processes such as phosphorylation become critical pathways in modulating caspase-8 activity [263–265]. The apoptotic capacity of caspase-8 can be modulated by phosphorylation of specific residues on the protein, such as tyrosine residues 397, 380, and 465, and serine residues 387 and 305 [266–268]. The phosphorylation of caspase-8 on tyrosine residues, unique among caspases [186], can alter its catalytic activity either by changing substrate recognition or by disrupting the active site [239]. During the formation of the DISC, Polo-like kinase 3 (Plk3) can phosphorylate the threonine residue T273 on its substrate, procaspase-8, thereby exerting a pro-apoptotic effect [266]. Phosphorylation at Tyr-310 facilitates dephosphorylation of caspase-8 by tyrosine phosphatase-1 (SHP-1), thereby promoting apoptosis. Conversely, phosphorylation of tyrosine residues Tyr-397 and Tyr-465 by the nonreceptor tyrosine kinase Lyn can inhibit caspase-8 cleavage and activation, thereby inhibiting the apoptotic process [186]. Regulation of caspase-8 also involves the maturation of its precursor, with some studies indicating that phosphorylation of procaspase-8 by Src kinase reduces its maturation rates [269, 270]. Further, serine phosphorylation is implicated in the regulation of caspase-8, with its serine residues being phosphorylated by ERK1/2 and CDK1 to prevent apoptosis [266–268, 271].

Beyond the extensively studied phosphorylation, ubiquitination emerges as a key mechanism in regulating caspase-8 activity. Ubiquitination, another form of PTM, involves the covalent attachment of ubiquitin (Ub) to target proteins [261]. This regulatory process plays a pivotal role in modulating apoptosis and necroptosis pathways, potentially offering new therapeutic avenues for treating cancer [272] and neurological disorders [273, 274]. Caspase-8 has been identified as a target for ubiquitination by the linear ubiquitin chain assembly complex (LUBAC) following TRAIL stimulation, consequently resulting in its inhibition [275]. Moreover, as previously mentioned, FLIP plays an indispensable role in regulating caspase-8. FLIP_L/S_ can form heterodimers with procaspase-8, where both are traditionally thought to inhibit caspase-8 activation. However, depending on its relative levels, FLIP_L_ may exhibit catalytic activity (albeit spatially constrained [275]) within procaspase-8 heterodimers and can facilitate caspase-8 activation [8, 275–280]. Like caspase-8, the expression of FLIP is also stringently regulated by ubiquitination [281–283]. In addition, within the cell death pathways regulated by caspase-8, other components such as FADD [284], RIPK1 [285–287], RIPK3 [288–293], and MLKL [293, 294] are also subject to regulation by ubiquitination, suggesting that ubiquitination could be a vital method in controlling caspase-8 expression and its mediated cell death pathways. Beyond phosphorylation and ubiquitination, nitric oxide (NO) has been shown to protect hepatocytes from TNF-α/ActD-induced apoptosis by interrupting the mitochondrial apoptosis signaling through *S*-nitrosylation of caspase-8 [262]. These studies suggest that the PTMs associated with caspase-8 are more complex than previously thought, providing greater opportunities to investigate and address unknown medical challenges by targeting caspase-8, the switch determining cellular fate. The related modifications and regulation types of caspase-8 are shown in Table 2.

**Table 2:** Caspase-8 related modifications and regulatory types

Modification type	Specific site	Functional impact	Associated conditions or processes	References
Methylation	CpG sites in promoter	Suppresses mRNA expression	Hepatocellular carcinoma, malignant gliomas	[259, 260]
Phosphorylation	Tyrosine residues: Y397, Y380, Y465; serine residues: S387, S305	Modulates apoptotic capacity	Apoptosis regulation	[239, 266–268]
	Threonine residue: T273 (by Plk3)	Pro-apoptotic effect	DISC formation, apoptosis	[266]
	Tyrosine residues: Y397, Y465 (by Lyn)	Inhibits caspase-8 cleavage and activation	Apoptosis inhibition	[185]
	Tyrosine residues: Y397, Y465, Y380 (by Src kinase)	Reduces procaspase-8 maturation rates	Precursor maturation	[259, 260]
	Serine residues: S387, S305 (by ERK1/2 CDK1, Plk1)	Prevents apoptosis	Apoptosis inhibition	[266–268, 271]
Dephosphorylation	Tyrosine residue: Y310 (by SHP-1)	Promotes apoptosis	Apoptosis regulation	[239]
Ubiquitination	Not specified	Modulates activity, inhibits caspase-8	Apoptosis, necroptosis, cancer, neurological disorders	[261, 272–275]
*S*-nitrosylation	Not specified	Protects against TNF-a/ActD-induced apoptosis	Hepatocyte protection, apoptosis inhibition	[262]

### Dual roles of caspase-8 inhibition and activation in disease treatment

Caspase-8 has emerged as a critical target for the therapeutic intervention in various diseases associated with cell death. Numerous molecules regulating caspase-8 have been developed or investigated. In recent years, caspase-8 inhibitors have gained significant attention for their ability to control caspase-8 mediated cell death or other physiological functions. Caspase-8 inhibitors can be broadly categorized into broad-spectrum caspase inhibitors and caspase-8-specific inhibitors. The broad-spectrum caspase inhibitors include zVAD-fmk [295–297], qVD-OPh (QVD) [295, 298], VX-166 [299], and cytokine response modifier A (CrmA, a viral inhibitor capable of suppressing both caspase-1 and caspase-8) [300]. On the other hand, caspase-8-specific inhibitors primarily consist of zIETD-fmk [297, 301, 302], Ac-IETD-CHO [303, 304], and several potential zinc compounds, including ZINC19370490 [305] and ZINC38200481 [151]. Studies have revealed that inhibiting caspase-8 with zVAD-fmk or zIETD-fmk can induce necroptotic death in activated microglia, thereby protecting neurons in the brain from inflammatory damage [301]. This suggests that caspase-8 inhibitors may serve as potential targets for treating neurological diseases. Furthermore, the application of caspase-8 inhibitors has also been discovered in research related to sepsis treatment. Researchers found that the caspase-8-specific inhibitor, zIETD-fmk, could partially reduce the activation of monocytes during sepsis, while caspase-8 inhibition could also induce necroptotic death in activated monocytes [302]. Moreover, Hotchkiss and colleagues have confirmed that intraperitoneal injection of the caspase inhibitor QVD can enhance the survival rate of mice with septic shock while reducing levels of pro-inflammatory cytokines [306]. Similarly, Weber et al. discovered that the caspase inhibitor VX-166 produced effects comparable to QVD, exerting positive action in both endotoxin shock and cecal ligation and puncture models of sepsis, while lowering levels of pro-inflammatory cytokines [299]. These studies suggest that caspase-8 inhibitors may represent an alternative strategy for treating sepsis, offering an important approach to combating the severe inflammatory responses associated with sepsis. Additionally, in the field of tumor therapy, zVAD-fmk, zIETD-fmk, and CrmA have been found to block TNFR2-mediated apoptosis in rat/mouse T-cell hybridoma PC60 cells, potentially offering a novel avenue for cancer treatment [297]. Given the potential therapeutic role of caspase-8 in inflammatory diseases, developing targeted caspase-8 inhibitory drugs is deemed necessary. Currently, specific inhibitors of caspase-8 are scarce. Thus, to expand the repertoire of caspase-8 inhibitors, scholars have employed specific algorithms to search for potential caspase-8 inhibitors within zinc compound libraries. Ahmad et al. [305] screened the zinc database and identified ZINC19370490 and ZINC04534268 as candidate compounds, while Jamal et al. [151] also screened five zinc compounds, including ZINC38200481. Although the effects of these compounds have yet to be validated, such research provides new insights into the discovery of caspase-8 inhibitors.

Relative to caspase-8 inhibitors, direct agonists targeting caspase-8 are exceedingly rare, likely due to the critical role of caspases in cellular apoptosis. Consequently, most interventions focus on inhibiting, rather than enhancing, the activity of caspase-8. Molecules that positively regulate caspase-8 are primarily utilized in cancer research, owing to the beneficial effects of caspase-8-mediated cell death in eliminating tumor cells. Second mitochondria-derived activator of caspase (Smac) mimetics are capable of initiating apoptosis via caspase-8 activation, thereby exhibiting antineoplastic properties [307, 308]. For instance, Servida et al. revealed that Smac mimetics impeded the function of inhibitor of apoptosis proteins (IAPs) in hematologic malignancies, consequently leading to the apoptotic death of neoplastic cells. Furthermore, these mimetics have been observed to work synergistically with cytarabine, etoposide, and notably with TRAIL in combinatory treatments, indicating that Smac mimetics may usher in a new era of anticancer therapeutics with considerable potential [307]. In addition, both bortezomib (PS-341) and the immunomodulatory agent lenalidomide have been demonstrated to activate caspase-8, thereby inducing apoptosis in myeloma cells. This approach offers a promising strategy for curtailing myeloma cell proliferation and enhancing apoptotic processes [309, 310]. Previous discussion on the pivotal role of caspase-8 activation in chemotherapeutic regimens for cancer treatment underscores the strategic importance of targeting caspase-8 to overcome the evasion of tumor cell death. Chemotherapeutic agents such as paclitaxel [311, 312], doxorubicin [313], and etoposide [313, 314] have been demonstrated to enhance caspase-8 activity and its consequent apoptosis, signifying a advancement for oncology treatments. For example, paclitaxel has been proven to potentiate caspase-8-mediated apoptosis through its interactions with microtubule-associated death effector domains [311], while doxorubicin and etoposide have been shown to significantly sensitize small cell lung cancer (SCLC) cells expressing caspase-8 to TRAIL-induced apoptosis [313]. These findings underscore the extensive prospects for targeted research on caspase-8 in the advancement of cancer therapy. Nonetheless, despite strides in foundational research, the intricate molecular mechanisms are yet to be fully elucidated, and the clinical application of related pharmaceuticals remains a challenge. An analysis of factors related to caspase-8 activity is delineated in Table 3.

**Table 3:** Caspase-8 related influencing factors

Negative effect on caspase-8	Mechanism	References
zVAD-fmk	zVAD-fmk is a pan-caspase inhibitor used to block apoptosis	[295–297]
QVD	QVD operates as a robust, irreversible inhibitor of broad-spectrum caspases, effectively thwarting apoptosis across diverse cell lines	[295, 298]
VX-166	VX-166 is a caspase inhibitor that inhibits the activity of caspase, to a certain extent playing the same role as QVD	[299]
CrmA	CrmA, a viral serpentine protein, inhibits certain cysteine proteases, such as caspase-1 and caspase-8	[300]
zIETD-fmk	zIETD-fmk is a selective caspase-8 inhibitor, commonly used in research to block the enzyme’s activity	[297, 301, 302]
Ac-IETD-CHO	Ac-IETD-CHO is a selective caspase-8 inhibitor that can be used to selectively inhibit caspase-8	[303, 304]
ZINC19370490	ZINC19370490 refers to a specific chemical compound listed in the ZINC database. It may be a potent caspase-8 inhibitor, but it is not yet proven	[305]
ZINC04534268	ZINC04534268 refers to a specific chemical compound listed in the ZINC database. It may be a potent caspase-8 inhibitor, but it is not yet proven	[305]
ZINC38200481	ZINC38200481 refers to a specific chemical compound listed in the ZINC database. It may be a potent caspase-8 inhibitor, but it is not yet proven	[151]
ZINC01576107	ZINC01576107 refers to a specific chemical compound listed in the ZINC database. It may be a potent caspase-8 inhibitor, but it is not yet proven	[151]
ZINC02384806	ZINC02384806 refers to a specific chemical compound listed in the ZINC database. It may be a potent caspase-8 inhibitor, but it is not yet proven	[151]
ZINC38570006	ZINC38570006 refers to a specific chemical compound listed in the ZINC database. It may be a potent caspase-8 inhibitor, but it is not yet proven	[151]
ZINC38569951	ZINC38569951 refers to a specific chemical compound listed in the ZINC database. It may be a potent caspase-8 inhibitor, but it is not yet proven	[151]
*Positive effect on caspase-8*		
(Smac) mimetics	(Smac) mimetics refer to small-molecule inhibitors that mimic the action of the second mitochondria-derived activator of caspases (Smac), targeting proteins that inhibit apoptosis, thereby promoting the activation of caspase-8 and subsequent induction of apoptosis	[307, 308]
PS-341	PS-341 can activate caspase-8 and promote apoptosis	[309, 310]
Lenalidomide	Lenalidomide can activate caspase-8 and promote apoptosis	[309, 310]
Paclitaxel	Paclitaxel can facilitate the activation of caspase-8 and thereby inducing apoptosis mediated by this enzyme	[311, 312]
Doxorubicin	Doxorubicin can facilitate the activation of caspase-8 and thereby inducing apoptosis mediated by this enzyme	[313]
Etoposide	Etoposide can facilitate the activation of caspase-8 and thereby inducing apoptosis mediated by this enzyme	[313, 314]

## Conclusions

Within the domain of biological research, caspase-8 stands out as a molecule of significant interest. As a member of the cysteine–aspartic acid protease family, caspase-8 has traditionally been recognized for its pivotal role in orchestrating cellular apoptosis. Recent investigations, however, have expanded our understanding, revealing caspase-8’s critical involvement in not only apoptosis but also in its intricate connections with pyroptosis and necroptosis, alongside its contributions to the activation and modulation of inflammasomes and inflammatory cytokines. Cell death is crucial in maintaining the balance of biological systems, with caspase-8 serving as a crucial modulator that significantly influences cellular destiny. Given its intricate role in cell death, we underscore the concept of PANoptosis, a form of cell death that integrates pyroptosis, apoptosis, and necroptosis, to better elucidate the often-ambiguous phenomena of cellular mortality in disease progression. The activity and expression levels of caspase-8 are intimately associated with the onset and progression of various diseases, rendering it a potential molecular target.

A vast array of studies has delineated a strong link between the aberrant expression or functional anomalies of caspase-8 and the emergence and progression of various inflammatory diseases, including immune system disorders, NDDs, sepsis, and cancer. Hence, a profound comprehension of the association between caspase-8 dysregulation and these diseases holds significant implications for their diagnosis, prevention, and therapeutic intervention. This review aims to summarize the research progression on caspase-8 across several pivotal diseases, accentuating three distinct characteristics of caspase-8 in the context of disease pathogenesis and therapeutics: firstly, the broad spectrum of caspase-8’s influence spans across apoptosis, pyroptosis, and necroptosis, implicating it in diverse cell death signaling cascades and in the pathophysiology of numerous ailments. Secondly, the underlying mechanisms are complex, as the distinctive contributions of caspase-8 to cell death are challenging to discern, given the potential overlap of pyroptosis, apoptosis, and necroptosis in the cell death process. Concurrently, the regulation of caspase-8 involves an extensive array of upstream and downstream molecules, with the precise molecular mechanisms yet to be fully elucidated. Thirdly, the outlook for future research is promising; the identification and development of novel therapeutic drugs targeting caspase-8 represent a promising direction. Interfering with the caspase-8 pathway could facilitate targeted treatments and management of specific diseases, expanding the therapeutic repertoire for clinical application. Moreover, further exploration into the roles and regulatory mechanisms of caspase-8 in cell death and diseases is necessary, aiming to clarify its differential functions across various cell types and organ systems, and to expedite the development of drugs targeting caspase-8, thereby achieving precise modulation of cell death pathways. While current research on the relationship between caspase-8 and cell death is predominantly experimental, the initiation of clinical studies and trials represents an uncharted territory that warrants exploration. Given the constraints of space and the specific focus of this article, our discussion primarily centers on caspase-8. Nevertheless, it is important to acknowledge that other members of the caspase family also hold significant potential as therapeutic targets. For example, caspase-10 plays a critical role in mediating various cell death pathways, including apoptosis, pyroptosis, and necroptosis [315, 316]. Research indicates that in human macrophages, caspase-10 exhibits greater proteolytic activity than caspase-8 during RIPK1 cleavage and shows an enhanced capacity to form complexes with RIPK1 and FADD [315]. Furthermore, variations in caspase-10 activity may be implicated in the onset and progression of various diseases. For instance, inadequate caspase-10 activity may result in the failure to eliminate malignant cells, thereby facilitating cancer progression [317, 318]. Despite its potential, research on caspase-10 remains relatively underdeveloped. We advocate for increased focus on the caspase family, given their extensive involvement in various cell death processes. A comprehensive understanding of cell death is essential to elucidating the pathogenesis and progression of numerous diseases, and the application of this knowledge to develop therapeutic interventions remains our ultimate objective.

## Data Availability

Data sharing not applicable to this article as no datasets were generated or analyzed during the current study. All information is derived from publicly available articles and datasets.

## Abbreviations

AA: Acetic acid
AD: Alzheimer’s disease
A2AR: Adenosine 2A receptor
AIH: Autoimmune hepatitis
AIM2: Absent in melanoma 2
ALPS: Autoimmune lymphoproliferative syndrome
ANXA1: Annexin A1
ASC: Apoptosis-associated speck-like protein containing a CARD
Aβ: Amyloid-beta
CARD: Caspase recruitment domain
CD: Crohn’s disease
cFLIP: Cellular FLICE-inhibitory protein
cIAP: Cellular inhibitors of apoptosis
CpG: Cytosine phosphate guanine
CrmA: Cytokine response modifier A
CYLD: Cylindromatosis
DAMPs: Damage-associated molecular patterns
DCs: Dendritic cells
DD: Death domain
DED: Death effector domain
DRs: Death receptors
DISC: Death-inducing signaling complex
FADD: FAS-associated protein with death domain
FASL: FAS ligand
Fbxo7: F-box only protein 7 gene
FLICE: FADD-like ICE
GBM: Glioblastoma multiforme
GSDMD: Gasdermin D
IAPs: Inhibitor of apoptosis proteins
IBD: Inflammatory bowel disease
IEC: Intestinal epithelial cell
LPS: Lipopolysaccharides
LUBAC: Linear ubiquitin chain assembly complex
MACH: MORT1-associated CED-3 homolog
Mch5: Mammalian Ced homolog 5
MR: Mendelian randomization
MS: Multiple sclerosis
NAIP5: Neuronal apoptosis inhibitory protein 5
NB: Neuroblastoma
NDDs: Neurodegenerative diseases
NFTs: Neurofibrillary tangles
NLRC4: NLR family CARD-containing protein 4
NLRs: Domain-like receptors
PARP: Polyadenosine diphosphate ribose polymerase
PAMPs: Pathogen-associated molecular patterns
PCD: Programmed cell death
PD: Parkinson’s disease
PDC: Pyruvate dehydrogenase complex
Plk3: Polo-like kinase 3
PNS: Peripheral nervous system
PRRs: Pattern recognition receptors
PTPN6: Protein tyrosine phosphatase nonreceptor type 6
QVD: QVD-OPh
RHIM: Receptor-interacting protein homotypic interaction motif
RIPK1: Receptor-interacting protein kinase 1
PYD: Pyrin domain
RA: Rheumatoid arthritis
SI–AKI: Sepsis-induced acute kidney injury
SCLC: Small cell lung cancer
Smac: Second mitochondria-derived activator of caspase
SS: Sjögren’s Syndrome
TICAM1: TIR domain-containing adapter molecule 1
TLR: Toll-like receptors
TME: Tumor microenvironment
TNFR1: Tumor necrosis factor receptor 1
TRADD: Tumor necrosis factor receptor type 1-associated death domain protein
Ub: Ubiquitin
UC: Ulcerative Colitis
ZBP1: Z-DNA binding protein 1
